# Transferrin Is Up-Regulated by Microbes and Acts as a Negative Regulator of Immunity to Induce Intestinal Immunotolerance

**DOI:** 10.34133/research.0301

**Published:** 2024-01-25

**Authors:** Xiaopeng Tang, Mingqian Fang, Ruomei Cheng, Junkun Niu, Xiaoshan Huang, Kuanhong Xu, Gan Wang, Yang Sun, Zhiyi Liao, Zhiye Zhang, James Mwangi, Qiumin Lu, Aili Wang, Longbao Lv, Chao Liu, Yinglei Miao, Ren Lai

**Affiliations:** ^1^ Engineering Laboratory of Peptides of Chinese Academy of Sciences, Key Laboratory of Bioactive Peptides of Yunnan Province, KIZ-CUHK Joint Laboratory of Bioresources and Molecular Research in Common Diseases, National Resource Center for Non-Human Primates, National Research Facility for Phenotypic & Genetic Analysis of Model Animals (Primate Facility), and Sino-African Joint Research Center, New Cornerstone Science Institute, Kunming Institute of Zoology, the Chinese Academy of Sciences, No.17 Longxin Road, Kunming, Yunnan, 650201, China.; ^2^ School of Basic Medicine, Qingdao University, Qingdao 266071, Shandong, China.; ^3^ Department of Gastroenterology, First Affiliated Hospital of Kunming Medical University, Yunnan Institute of Digestive Disease, Kunming 650032, Yunnan, China.; ^4^ Yunnan Province Clinical Research Center for Digestive Diseases, Kunming 650032, Yunnan, China.; ^5^ Kunming College of Life Science, University of Chinese Academy of Sciences, Kunming 650204, Yunnan, China.; ^6^ Hefei National Laboratory for Physical Sciences at the Microscale and School of Life Sciences, University of Science and Technology of China, Hefei 230027, Anhui, China.; ^7^ Center for Evolution and Conservation Biology, Southern Marine Science and Engineering Guangdong Laboratory, Guangzhou 511458, Guangdong, China.

## Abstract

Cross-talks (e.g., host-driven iron withdrawal and microbial iron uptake between host gastrointestinal tract and commensal microbes) regulate immunotolerance and intestinal homeostasis. However, underlying mechanisms that regulate the cross-talks remain poorly understood. Here, we show that bacterial products up-regulate iron-transporter transferrin and transferrin acts as an immunosuppressor by interacting with cluster of differentiation 14 (CD14) to inhibit pattern recognition receptor (PRR) signaling and induce host immunotolerance. Decreased intestinal transferrin is found in germ-free mice and human patients with ulcerative colitis, which are characterized by impaired intestinal immunotolerance. Intestinal transferrin and host immunotolerance are returned to normal when germ-free mice get normal microbial commensalism, suggesting an association between microbial commensalism, transferrin, and host immunotolerance. Mouse colitis models show that transferrin shortage impairs host’s tolerogenic responses, while its supplementation promotes immunotolerance. Designed peptide blocking transferrin–CD14 interaction inhibits immunosuppressive effects of transferrin. In monkeys with idiopathic chronic diarrhea, transferrin shows comparable or even better therapeutic effects than hydrocortisone. Our findings reveal that by up-regulating host transferrin to silence PRR signaling, commensal bacteria counteract immune activation induced by themselves to shape host immunity and contribute for intestinal tolerance.

## Introduction

The animal gastrointestinal tract is colonized by various microorganisms including bacteria, fungi, and viruses [1,2]. The largest population of intestinal microbiota is bacteria (commensal bacteria). The animal host and its intestinal microbial flora affect one another to function together as a complex ecologic system [3]. On the one hand, a healthy intestine has the ability to shape the microbiota and to limit the colonization of the intestinal tract by harmful bacteria through a symbiotic relationship [4,5]. Host colon mucus layers create a physical barrier, which is composed of mucin glycoproteins that effect host–microbial interactions by separating bacterial flora and intestinal epithelial cells [6,7]. Moreover, hosts secrete antibacterial factors such as defensins, C-type lectins, lysozyme, phospholipase A2, and secreted immunoglobulin A to regulate the microbiota’s growth [8,9]. On the other hand, the intestinal microbiota plays a crucial role in shaping immune functions of their hosts [10]. The intestinal microbiota stimulates the development of both local and systemic immunity of hosts [11]. Very down-regulated immune responses and much smaller lymphoid organs have been found in germ-free animals [12,13], while microbial colonization of the animals increases in the systemic immunological capacity by elevating immunoglobulin and antibodies levels and changing mucosal-associated lymphocyte tissues and cell populations [14–16]. One main characteristic feature of the intestine is its ability to maintain tolerance to microbial antigens, showing a symbiotic host relationship [10,17]. Long evolution by mutually adapting and selecting each other creates the close symbiosis of the microbiota and its hosts. The symbiotic relationship maintains a constant homeostasis by perfectly regulating the microbial load and the immune response generated against it in the healthy human intestine, while dysbiosis of intestinal flora may result in various pathological conditions [18–20].

Hosts recognize invading microorganisms and trigger the activation of innate immunity, which then leads to the development of antigen-specific adaptive immunity through recognizing microbial components by Toll-like receptors (TLRs) [21]. TLRs and their coreceptor cluster of differentiation 14 (CD14) are up-regulated and activated by microbe components to induce host immunity [22,23]. However, commensal bacteria appear to use a mechanism to enhance colonization of the gut and thereby establish host–microbial tolerance [2,24–27], but the mechanism is unknown. Here, we reported that although microbiome-derived products (e.g., lipopolysaccharide [LPS], lipoteichoic acid [LTA], and bacterial DNA) stimulate immunity by up-regulating and activating TLR signaling, they also up-regulate and beneficially use host transferrin as a negative regulator of the TLR signaling to establish host’s immune tolerance.

## Results

### Bacterial products from both pathogenic and probiotic bacteria up-regulate transferrin expression in immune and non-immune cells via NF-κB activation

**Fig. 1.**
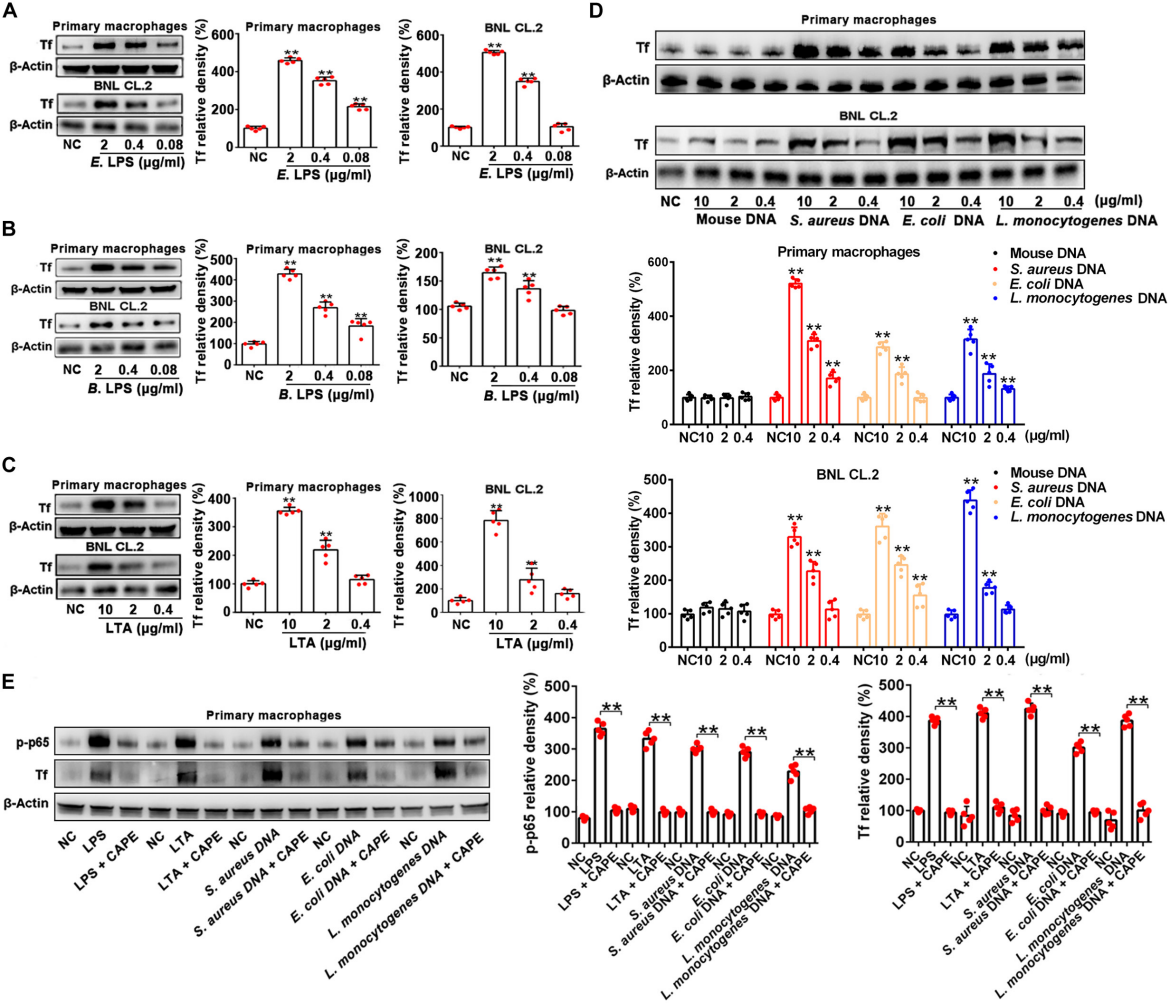
Bacterial products from both pathogen and probiotic bacteria up-regulate transferrin expression in immune and non-immune cells via NF-κB activation. (A to D) Effects of LPS from *E. coli* (*E*. LPS), LPS from *B. fragilis* (*B*. LPS), LTA from *S. aureus*, and bacterial DNAs treatment for 24 h on transferrin expression in primary macrophages and BNL CL.2 cells were analyzed by Western blotting. Corresponding quantifications are shown on the right (A to C) or at the bottom (D). Data represent means ± SD of 5 independent experiments, *P < 0.05, **P < 0.01 by one-way ANOVA with Dunnett’s post-hoc test. (E) NF-κB-regulated transferrin expression was inhibited by the specific NF-κB inhibitor caffeic acid phenethyl ester (CAPE, 20 μM, 30 min pretreatment). Transferrin (Tf) and phospho-p65 were analyzed by Western blotting. LPS (2 μg/ml), LTA (10 μg/ml), *E. coli* DNA (10 μg/ml), *S. aureus* DNA (10 μg/ml), or *L. monocytogenes* DNA (10 μg/ml) was used to promote Tf expression. Corresponding quantifications are shown on right. β-Actin was used as the control. Data represent means ± SD of 5 independent experiments, **P < 0.01 by unpaired *t* test. Tf, transferrin; CAPE, caffeic acid phenethyl ester.

As literatures reported, microbial infections cause iron deficiency anemia (IDA) and IDA promotes transferrin expression [28–30]. We tested effects of microbial products from both pathogenic and probiotic bacteria on transferrin expression in human primary macrophages and mouse normal embryonic liver cell line (BNL CL.2) to investigate the association of transferrin with bacterial infection and/or commensalism (Fig. 1). Western blot analysis (Fig. 1A), quantitative real-time polymerase chain reaction (qRT-PCR, Fig. S1A and B), and enzyme-linked immunosorbent assay (ELISA, Fig. S1C and D) indicated that transferrin was up-regulated by LPS from conditional pathogen bacterium *Escherichia coli* or probiotic bacterium *Bacteroides fragilis* (Fig. 1B), LTA from *Staphylococcus aureus* (Fig. 1C and Fig. S2), and bacterial DNA from *E. coli*, *S. aureus*, and *Listeria monocytogenes* free from enterotoxin contamination (Fig. 1D and Fig. S3) in a dose-dependent manner in all the cells.

Bacterial DNA containing multiple CpG nucleotides display stronger inflammatogenic properties than eukaryotic nuclear DNA [31,32]. As a comparison, mouse DNA, which contains fewer unmethylated CpG repeats and less inflammatogenic properties than bacterial DNA, showed little effect on transferrin expression in those cells (Fig. 1D), suggesting that the up-regulation of transferrin is involved in inflammatory response-induced bacterial products, in which NF-κB plays a central role. In addition, bacterial pathogens, *E. coli* and *S. aureus*, also up-regulate transferrin expression (Fig. S4). We next investigated the effects of the specific NF-κB inhibitor caffeic acid phenethyl ester (CAPE) on transferrin expression promoted by bacterial products. As illustrated in Fig. 1E, all the transferrin up-regulation induced by LPS, LTA, and bacterial DNA was blocked by CAPE in primary macrophages, indicating that transferrin up-regulation induced by bacterial products was via NF-κB activation.

### Transferrin binds to CD14 with high affinity

**Fig. 2.**
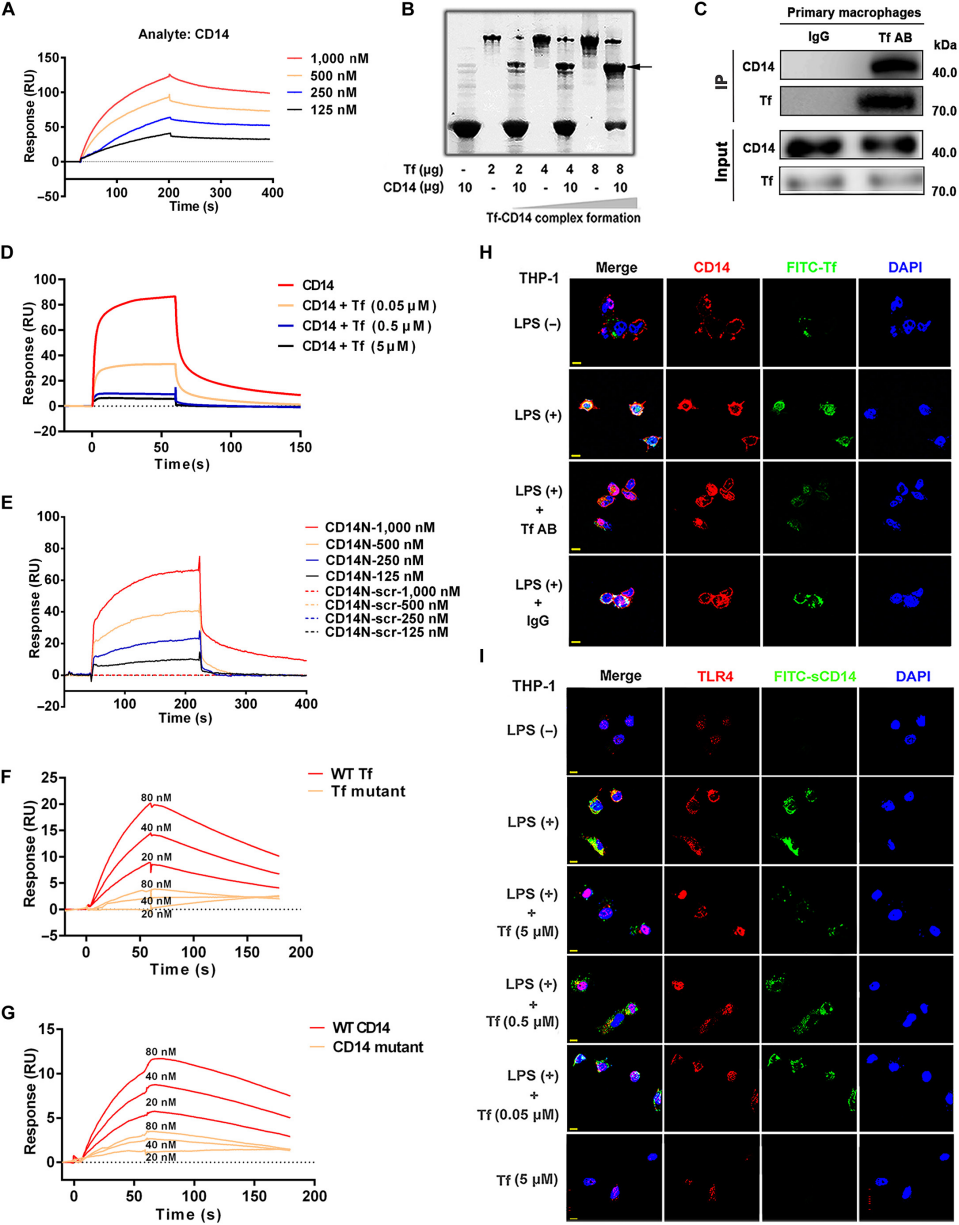
Direct in vitro and in vivo interactions between transferrin and CD14. (A) SPR analysis of transferrin–CD14 interaction. (B) Native gel shift analysis of transferrin–CD14 complex formation. (C) Coimmunoprecipitation of transferrin and CD14 in primary macrophages. (D) Inhibitory effects of transferrin on LPS–CD14 interaction by SPR analysis. (E) SPR analysis of interaction between transferrin and LPS binding region of CD14 N-terminus (CD14N) and CD14N-scr (scrambled control of CD14N). (F) SPR analysis of interaction between wild-type transferrin (WT-Tf) or transferrin mutants (R663A and K664A, Tf mutant) and CD14. (G) SPR analysis of interaction between transferrin and wild-type CD14 (WT-CD14) or CD14 mutants (D44A, S46A, and Q50A). (H) LPS increased CD14 expression and CD14-FITC-labeled transferrin (FITC-Tf) colocalization, which was inhibited by transferrin antibody (Tf AB), on THP-1 cell membranes, observed by confocal microscopy. (I) Transferrin interfered with TLR4-FITC-labeled CD14 colocalization induced by LPS on THP-1 cell membranes, observed by confocal microscopy. Cell nuclei were labeled by DAPI. Scale bar represents 10 μm. All images are representative of at least 3 independent experiments. Tf, transferrin.

Mass spectrometry-based coimmunoprecipitation analysis was used to screen targets of transferrin in human peripheral blood mononuclear cells (PBMCs). Results showed that transferrin interacted with CD14 directly (Fig. S5), as confirmed by surface plasmon resonance (SPR) (Fig. 2A) and native-polyacrylamide gel electrophoresis (Fig. 2B). The association (*K*_a_), dissociation (*K*_d_), and equilibrium dissociation constants (*K*_D_) of the interaction between transferrin and CD14 were 2.4 × 10^4^ M^−1^ s^−1^, 3.3 × 10^−4^ s^−1^, and 14 nM, respectively, thus showing high affinity (Fig. 2A). The interactions between transferrin and CD14 were further proved by using coimmunoprecipitation analysis (Fig. 2C) with comparative human serum albumin (HSA), which showed no interaction with CD14 (Fig. S6). Given the involvement of transferrin expression in NF-κB activation, and that many pathogen-associated molecular patterns (PAMPs) are recognized and presented by CD14 to activate NF-κB [33], transferrin–CD14 interaction may affect the recognition of PAMPs, such as LPS, by CD14.

### Transferrin blocks the binding and presentation of CD14 on LPS

CD14 is critical in the signaling pathways of several TLRs [34,35]. Notably, CD14 transfers LPS molecules to the TLR4/MD-2 complex to facilitate LPS recognition by TLR4 [36–39] and controls LPS-induced internalization of TLR4 [40,41]. We next investigated whether transferrin interferes with CD14–LPS interaction. As illustrated in Fig. 2D, the binding between CD14 and LPS was blocked by transferrin. The LPS-binding pocket is located in the N-terminal hydrophobic region of CD14 [42,43]. Here, the docking model of the transferrin–CD14 complex (Fig. S7) suggested that the LPS binding region (CD14N: E​LDD​EDF​RCV​CNF​SEP​QPD​WSE​AFQ​CVS​AVE​VEIHAGGLN) was responsible for the transferrin–CD14 interaction, as confirmed by SPR analysis (Fig. 2E). We identified 2 key residues of transferrin (R663 and K664) that likely play key roles in transferrin–CD14 interactions. Thus, transferrin mutants (R663A and K664A) were constructed (Fig. S8A, B, and E). Notably, the mutants exhibited weak interactions with CD14 (Fig. 2F). Furthermore, the docking model suggested that 3 key residues of the LPS-binding region of CD14 (D44, S46, and Q50) may participate in the transferrin–CD14 interaction. Compared with wild-type CD14 (Fig. 2G), the corresponding mutants (D44A, S46A, and Q50A) of CD14 (Fig. S8C to E) exhibited weak interactions with transferrin.

Given that LPS up-regulates the expression of CD14, TLRs [22,23], and transferrin (Fig. S1 and Fig. 1), we next investigated whether transferrin is colocalized with TLR4 or CD14 on cell membranes and whether the colocalizations are enhanced by LPS. As reported previously [22,23], LPS stimulation for 30 min increased the expression of CD14 on the cell membranes of the human monocytic cell line (THP-1) and the colocalization of FITC-labeled transferrin and CD14, while application of the transferrin antibody decreased the colocalization (Fig. 2H), further confirming direct CD14–transferrin interaction. Similarly, LPS also up-regulated TLR4 expression on the membranes of the cells and thus promoted the colocalization of TLR4 with FITC-labeled soluble CD14 (sCD14), while transferrin interfered with the colocalization (Fig. 2I). Notably, at the 5 μM dosage, transferrin diminished most of the TLR4-sCD14 colocalization.

### Transferrin inhibits TLR4 activation induced by LPS

**Fig. 3.**
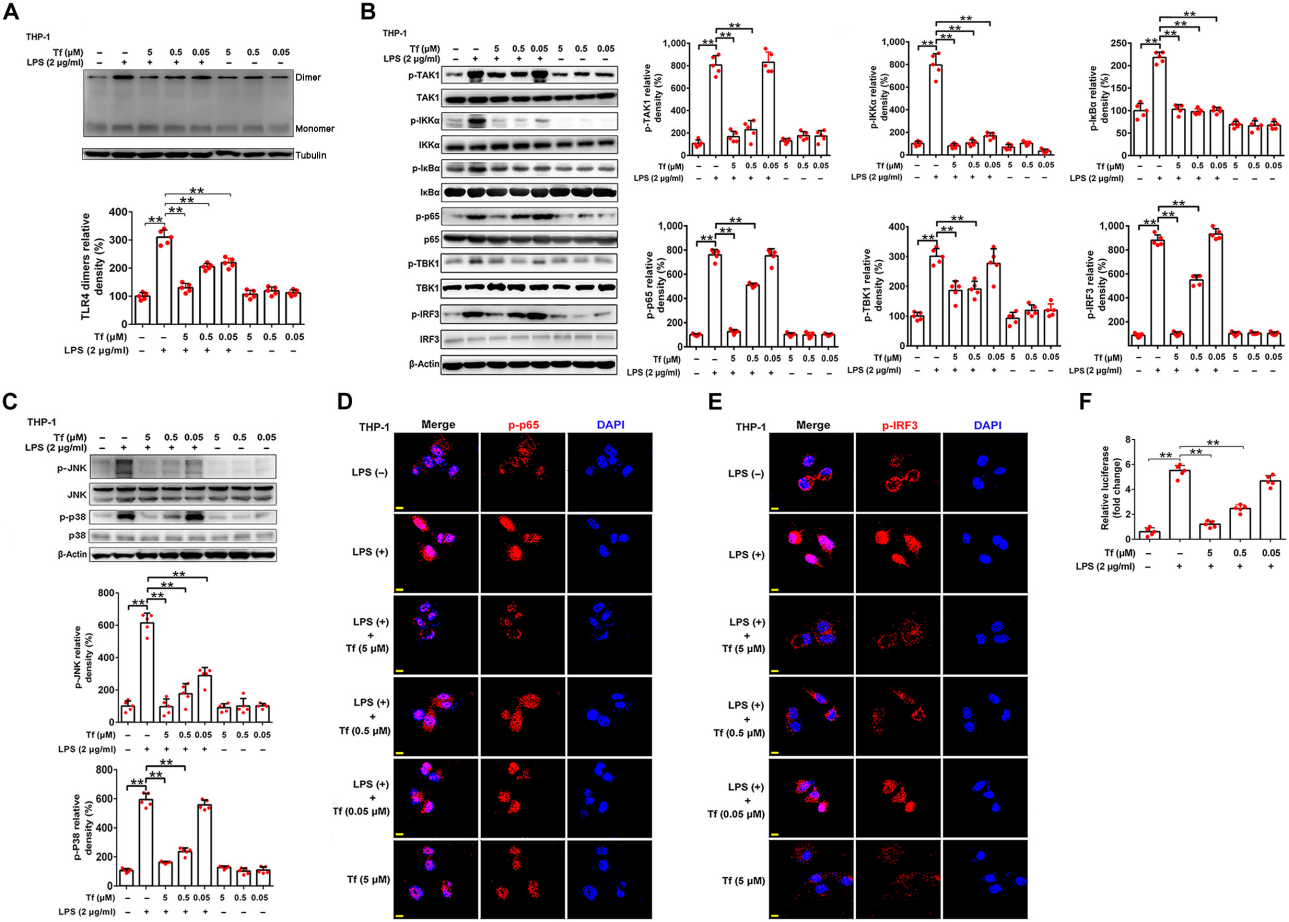
Transferrin inhibits TLR4 activation. (A) TLR4 dimerization in THP-1 cells induced by LPS was inhibited by transferrin analyzed by Western blotting. Corresponding quantifications are shown below. Tubulin was used as a loading control. (B) Western blotting analysis of transferrin’s inhibition on downstream signaling pathways (both myD88-dependent and -independent pathways) of TLR4 activation induced by LPS in THP-1 cells. TAK1, IKKα, IκBα, and p65 are involved in the myD88-dependent pathway. TBK1 and IRF3 are involved in the myD88-independent pathway. Corresponding quantifications are shown on the right. β-Actin was used as a loading control. (C) Western blotting analysis of transferrin’s inhibition on JNK and p38 phosphorylation induced by LPS in THP-1 cells analyzed by Western blotting. Corresponding quantifications are shown below. β-Actin was used as a loading control. (D and E) Transferrin inhibited nuclear translocation of p-p65 (D) and p-IRF3 (E) in THP-1 cells induced by LPS observed by confocal microscopy. Cell nuclei were labeled by DAPI. Scale bar represents 10 μm. Images are representative of at least 3 independent experiments. (F) Transferrin inhibited LPS-induced NF-κB-dependent reporter gene expression. Relative luciferase fold change was calculated. Data represent means ± SD of 5 independent experiments, *P < 0.05, **P < 0.01 by one-way ANOVA with Dunnett’s post-hoc test.

Based on CD14’s function transferring LPS to TLR4 and the current finding that transferrin interferes with interactions of CD14–LPS as above, we next tested if transferrin inhibited TLR4 activation induced by LPS. As illustrated in Fig. 3A, transferrin blocked LPS-induced TLR4 dimerization in THP-1 cells, which consequently decreased phosphorylation of transforming growth factor-β (TGF-β)-activating kinase 1 (TAK1), IκB (inhibitory subunit of nuclear factor κB [NF-κB]) kinase α (IKKα), IκBα, and NF-κB p65 of the myD88-dependent pathway and TRAF-associated NF-κB activator (TANK)-binding kinase 1 (TBK1) and interferon regulatory factor 3 (IRF3) of the myD88-independent pathway in THP-1 cells (Fig. 3B). Moreover, LPS-induced phosphorylation of JNK and p38 of the mitogen-activated protein kinase (MAPK) signaling pathway was also inhibited by transferrin in THP-1 cells (Fig. 3C). Confocal microscopy showed that nuclear translocations of p-p65 and p-IRF3 in THP-1 cells by LPS stimulation for 30 min (Fig. 3D and E) were also blocked by transferrin. In addition, NF-κB reporter assay showed that transferrin inhibited NF-κB activation induced by LPS in primary macrophages (Fig. 3F).

**Fig. 4.**
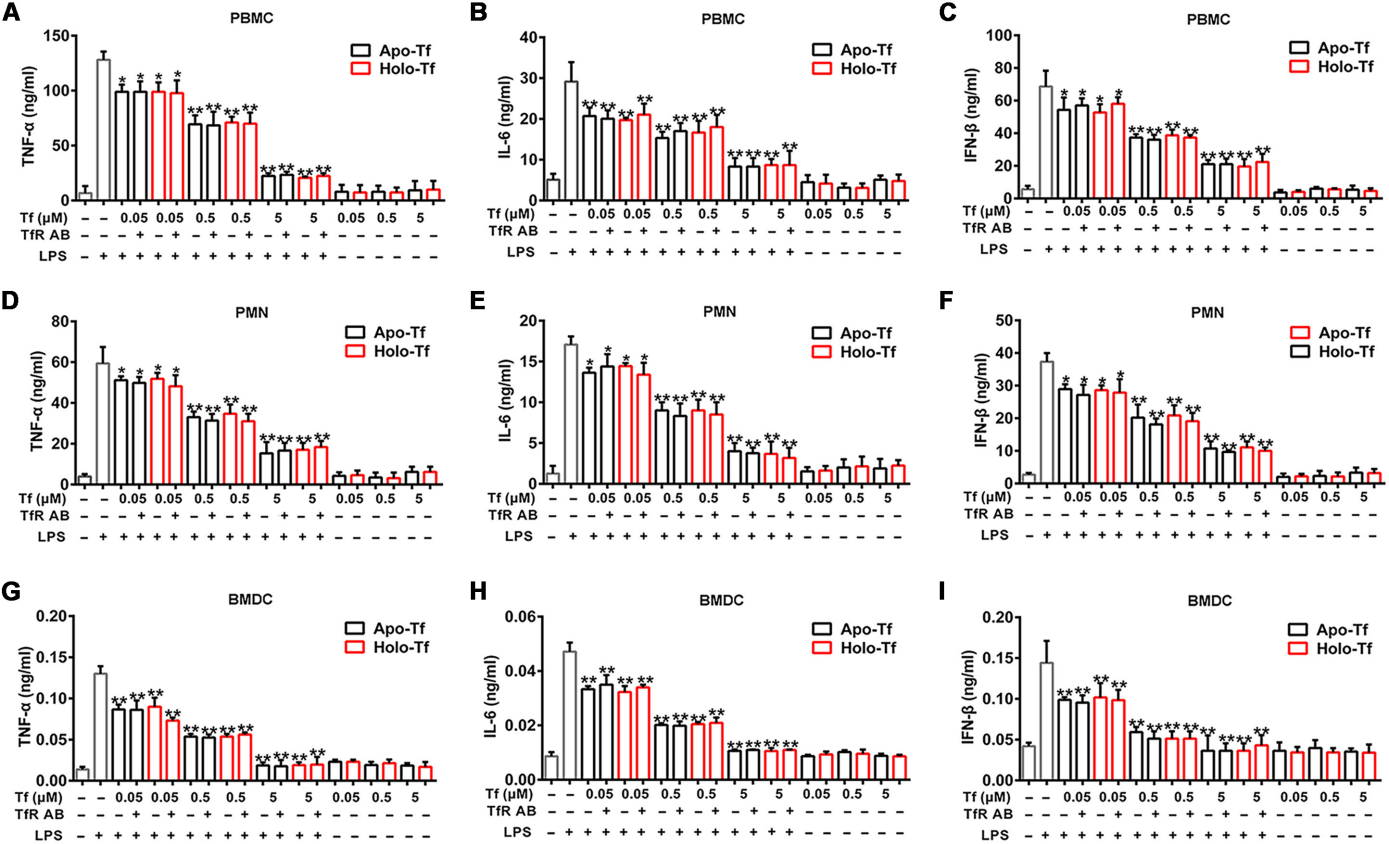
Transferrin inhibits production of cytokines and type I interferon induced by LPS. Primary PBMCs, primary PMNs, and primary mouse BMDCs were stimulated in the presence or absence of apo- or holo-transferrin by LPS for 8 h. Some groups of cells were first incubated with anti-transferrin receptor antibody (TfR AB, 10 μg/ml) for 30 min. Effects of apo- or holo-transferrin on TNF-α, IL-6, or IFN-β production induced by LPS in PBMCs (A to C), PMNs (D to F), or BMDCs (G to I) are shown. Data represent means ± SD of 5 independent experiments, *P < 0.05, **P < 0.01 by one-way ANOVA with Dunnett’s post-hoc test. Tf, transferrin.

TLR4 stimulation by LPS induces the release of many cytokines to activate potent immune responses [44]. Given transferrin’s inhibition on TLR4 activation evoked by LPS, we investigated its effects on cytokine release stimulated by LPS. As an iron carrier, transferrin exists in plasma in both the ferric iron-bound (holo-transferrin) and unbound states (apo-transferrin). As illustrated in Fig. 4 and Fig. S10A to F, both apo- and holo-transferrin showed similar inhibition on LPS-induced production of TNF-α, IL-6, IFN-β, or TGF-β in human PBMCs, polymorphonuclear neutrophils (PMNs), mice bone marrow dendritic cells (BMDCs), THP-1 cells, and HUVECs in a dose-dependent manner. Importantly, the blockage of transferrin-transferrin receptor (TfR) interaction using anti-TfR antibody (TfR AB) had no effect on the inhibition of elicited by transferrin, suggesting that the inhibition was independent of TfR.

To prove that transferrin–CD14 interaction is responsible to drive the phenotype seen in the subsequent cell and animal models, we designed a peptide TC6 (TTPEPC) that specifically inhibits transferrin–CD14 interaction (Fig. S9A) without affecting LPS’s binding to CD14 (Fig. S9B) and activation on TLR4 (Fig. S9A and B and Fig. 4P and Q), based on the structure of the LPS–CD14 complex [43,45] and the docking model of the transferrin–CD14 complex (Fig. S7). TC6 showed a high affinity (*K*_D_ 13 nM) with transferrin (Fig. S9C) and no interaction with CD14 and LPS (Fig. S9D and E). As expected, transferrin’s inhibition on TNF-α and IFN-β release induced by LPS in primary macrophages was blocked by TC6 (Fig. S10G and H).

### Transferrin overexpression and knockdown attenuates and aggravates inflammatory response induced by LPS, respectively

**Fig. 5.**
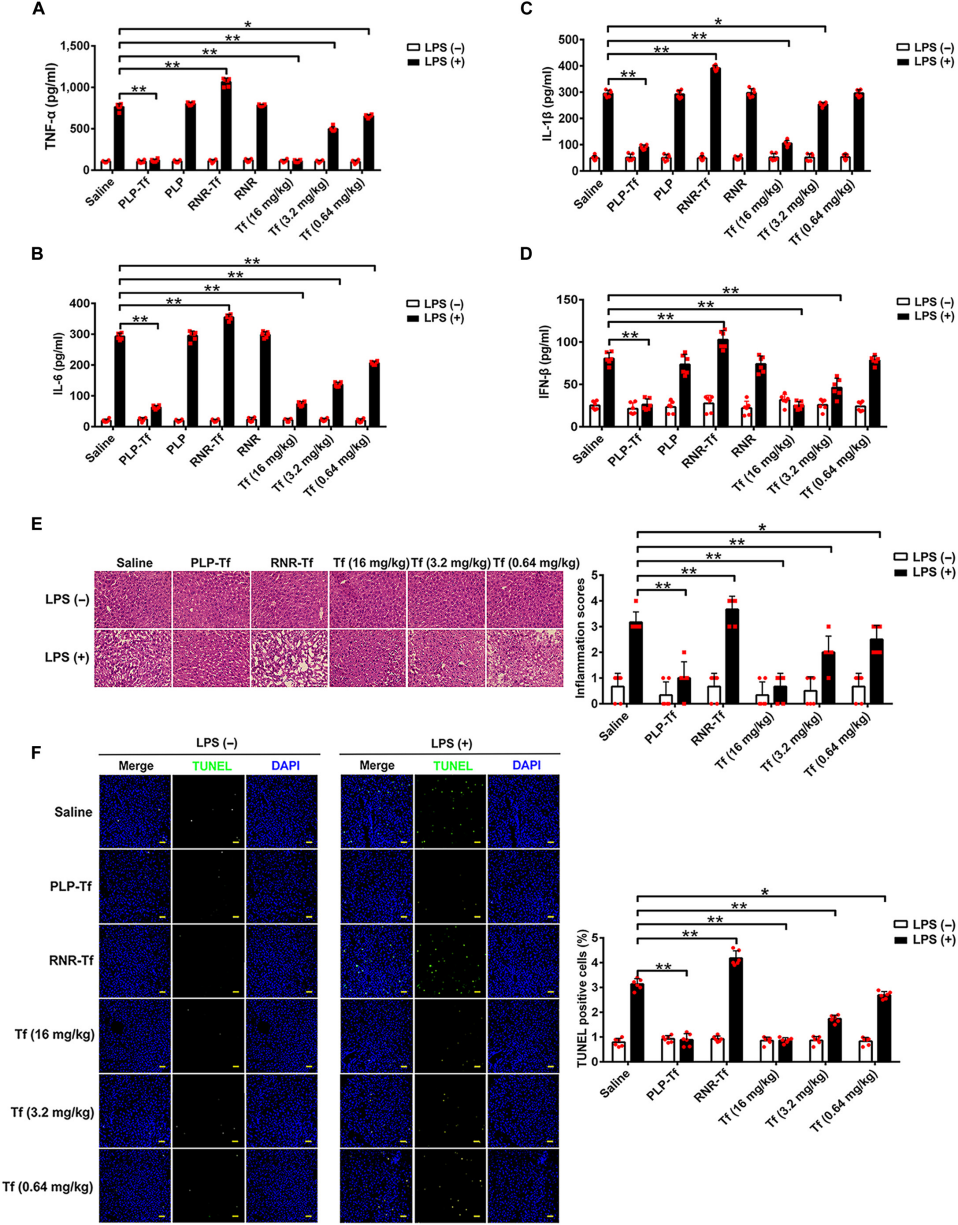
Transferrin inhibits inflammatory responses induced by LPS in vivo*.* LPS (750 μg/kg) was injected into the tail vein of mouse groups including transferrin overexpression (PLP-Tf), knockdown (RNR-Tf), or their blank (PLP and RNR), to induce an inflammatory response for 2 h. In the transferrin-treated group, LPS injection was performed after transferrin administration through the tail vein for 20 min. Plasma TNF-α (A), IL-6 (B), IL-1β (C), and IFN-β (D) levels were determined by ELISA. (E) LPS-induced liver injuries in all mouse groups were determined by hematoxylin and eosin (H&E) staining and corresponding quantifications are shown on the right. (F) Apoptosis induced by LPS was evaluated using an apoptosis detection kit and corresponding quantifications are shown on the right. Data represent means ± SD (*n* = 6), *P < 0.05, **P < 0.01 by one-way ANOVA with Dunnett’s post-hoc test. Tf, transferrin.

Transferrin overexpression (PLP-Tf) and knockdown mice (RNR-Tf) were used to further elucidate the role of transferrin in inflammatory responses (Fig. S11). As illustrated in Fig. 5A to D, the plasma levels of inflammatory factors (TNF-α, IL-6, IL-1β, and IFN-β) induced by LPS were decreased by both transferrin overexpression and intravenous injection of exogenous transferrin, but were exacerbated by transferrin knockdown. Effects of transferrin on inflammatory injury induced by LPS were evaluated by histological examination (Fig. 5E and F). Transferrin overexpression and exogenous transferrin administration alleviated LPS-induced liver injury, whereas the injury was aggravated by transferrin knockdown (Fig. 5E). Transferrin overexpression and exogenous transferrin administration inhibited LPS-induced apoptosis, while transferrin knockdown promoted it (Fig. 5F). As illustrated in Fig. S12A and B, plasma levels of alanine transaminase and aspartate aminotransferase up-regulated by LPS were decreased by both transferrin overexpression and intravenous injection of exogenous transferrin, but were exacerbated by transferrin knockdown. Importantly, transferrin overexpression, intravenous injection of exogenous transferrin, or transferrin knockdown showed no effects on iron level of plasma and liver tissue (Fig. S12C and D). In addition, transferrin overexpression and exogenous transferrin administration alleviated LPS or bacteria-induced mice lethality, whereas the lethality was aggravated by transferrin knockdown (Fig. S12E and F). As expected, the interference peptide TC6 blocked the transferrin’s inhibition on plasma TNF-α and IFN-β secretion in the mouse model (Fig. S13).

### Transferrin down-regulation impairs host tolerogenic responses

**Fig. 6.**
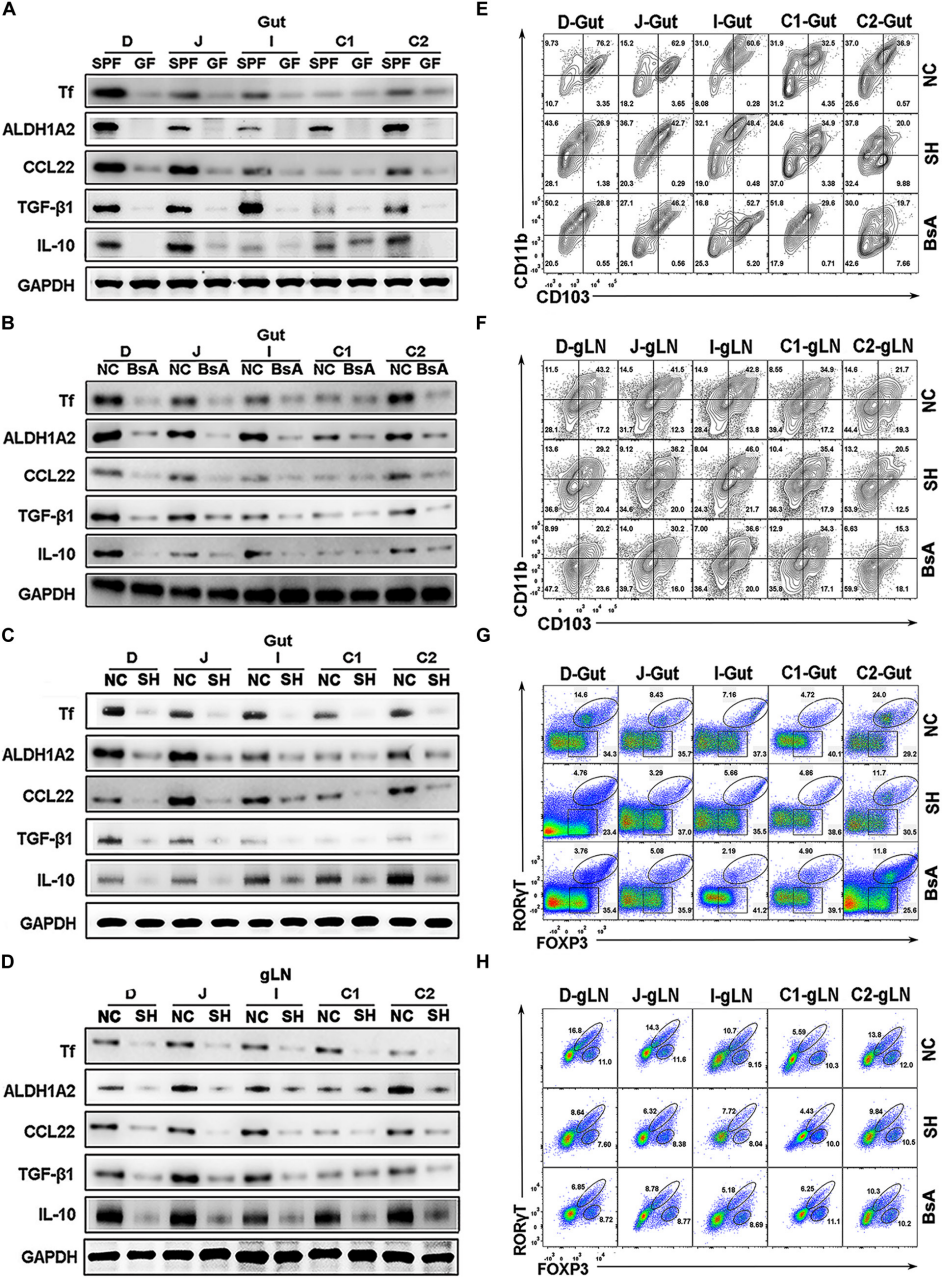
Transferrin shortage impairs host tolerogenic responses. (A and B) Transferrin, ALDH1A2, CCL22, TGF-β, and IL-10 levels in different gut tissue segments (duodenum, jejunum, ileum, cecum, and colon [D, J, I, C1, and C2] of SPF (NC), GF, and broad-spectrum antibiotic (BsA)-treated SPF mice were determined by Western blotting. GAPDH was used as the control. (C and D) Transferrin, ALDH1A2, CCL22, TGF-β, and IL-10 in different segments of gut tissue (duodenum [D], jejunum [J], ileum [I], cecum [C1], and colon [C2]) and in gut-draining lymph nodes (gLN) of SPF mice (NC) and transferrin knockdown SPF mice (SH) were analyzed by Western blotting. Dendritic cells (DCs) in gut tissue (E) and gLN (F) of NC mice, SH mice, and BsA-treated mice were characterized as CD45.2^+^MHCII^+^CD11c^+^ (Fig. S15A) and further subdivided into CD103^+^ DCs, CD103^+^CD11b^+^ DCs [double positive (DP) DCs], and CD11b^+^DCs. Tregs in gut tissue (G) and gLN (H) of all mice groups were characterized as CD45.2^+^CD4^+^ (Fig. S15B) and further subdivided into Foxp3^+^ Tregs and Foxp3^+^RORγT^+^ Tregs. Tf, transferrin.

Transferrin showed marked ability to inhibit inflammation induced by LPS as above, suggesting its potential to promote host immune tolerance. Germ-free (GF) mice have little microbial commensalism and impaired immunotolerance [46,47]. We first compared transferrin levels in GF and specific pathogen-free (SPF) mice to investigate the association among microbial commensalism, transferrin level, and immunotolerance. The average transferrin concentration in the plasma of GF mice (*n* = 10; 5 male and 5 female) was 1.84 mg/ml (SD 0.32), whereas that in SPF mice (*n* = 10; 5 male and 5 female) was 2.237 mg/ml (SD 0.27) (Fig. 6A). Decreased levels of transferrin were also observed in the liver and spleen of GF mice (Fig. S14). Plasma and tissue transferrin levels were returned to normal when the GF mice lived at an SPF environment for 4 weeks (Fig. S14).

As above, markedly down-regulated transferrin was observed in germ-free mice that share impaired immunotolerance, suggesting a possible association of transferrin down-regulation with immunotolerance. Given that GF mice are broadly impaired in many aspects of development and early immune education [48], we next compared the ability of intestinal tolerance in GF mice and in broad-spectrum antibiotics (BsA)-treated mice to specifically investigate the association of intestinal microbiota with transferrin and host–microbial homeostasis. As illustrated in Fig. 6A, transferrin levels in different segments of gut tissue (duodenum, jejunum, ileum, and colon [D, J, I, and C2]) of GF mice were markedly lower (~5- to 100-fold reduction) than those in SPF mice. This was consistent with the lower levels of aldehyde dehydrogenase 1 family member A2 (ALDH1A2), CCL22, TGF-β, and IL-10 observed in GF mice, which are known to play key roles in maintaining intestinal tolerance [49]. After treatment with BsA to deplete gut microbiota, decreases in transferrin and corresponding ALDH1A2, CCL22, TGF-β, and IL-10 levels were also observed in the gut of BsA-treated mice (Fig. 6B). No obvious change of transferrin and other factors related with intestinal tolerance was observed in the segment of cecum (C1), which shows little function in maintaining intestinal tolerance [49].

As illustrated in Fig. 6C and D, the levels of makers of immune tolerance including ALDH1A2, CCL22, TGF-β, and IL-10 [49,50] were decreased in the gut and gLN of transferrin-knockdown SPF mice, which were consistent with the decreased transferrin level. CD103^+^CD11b^+^ dendritic cells (DCs), regulatory T cells (Tregs), and regulatory B cells (Bregs) have been implicated in gut tolerogenic responses [49,50]. A gating strategy was used to analyze the DCs, Tregs, and Bregs subsets in intestinal tissues and lymph node (Fig. S15). As illustrated in Fig. 6E and F and Fig. S16, marked reductions in the frequency, number, and proliferation of CD103^+^CD11b^+^ DCs in the D-gut and D-gLN of both transferrin-knockdown and BsA-treated mice were observed. Similar decreased patterns were also observed for Foxp3^+^ Tregs, RORγT^+^ Tregs (Fig. 6G and H and Fig. S17), and CD19^+^CD5^+^Bregs (Fig. S18). Notably, Treg was decreased in D- and C2-gLN of TC6-treated SPF mice (Fig. S19).

### Protective effects of transferrin on intestinal immune imbalance induced by gut microbial dysbiosis

**Fig. 7.**
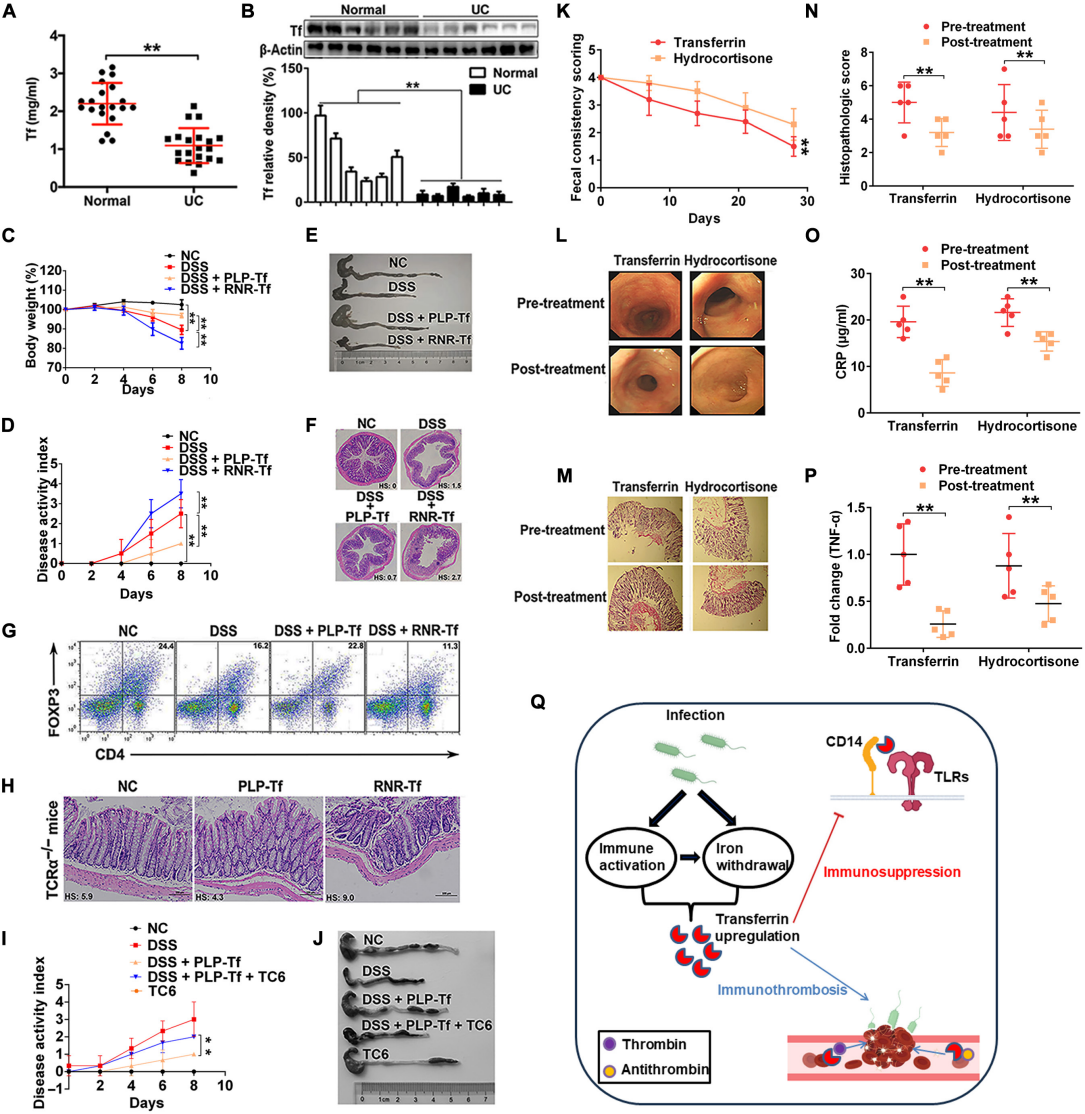
Transferrin supplementation promotes immune tolerance and intestinal homeostasis. (A) Amounts of transferrin in plasma from ulcerative colitis (UC) patients and healthy volunteers (Normal) were determined by ELISA. Data represent mean ± SD (*n* = 20), **P < 0.01 by unpaired *t* test. (B) Western blot analysis (top) and quantification (bottom) of transferrin in tissue samples from UC patients and healthy volunteers. β-Actin was used as a loading control. Data represent means ± SD (*n* = 6), **P < 0.01 by Kruskal–Wallis test followed by Bonferroni adjustment. Lentivirus for transferrin overexpression (PLP-Tf) and retrovirus for transferrin knockdown (RNR-Tf) were injected into C57BL/6 or TCRαKO mice (16 weeks old) through the tail vein to induce transferrin overexpression and knockdown, respectively. DSS (5%) was added to drinking water to induce acute colitis. Changes in body weight (%) (C), disease activity index (D), colon length (E), and colon histopathological injury (F) are shown. (G) Foxp3^+^ Tregs in gLN of the mouse DSS model were analyzed. Colon histopathological injury (H) at 20 weeks was assayed in TCRα KO mice with colitis. Effect of TC6 treatment (10 mg/kg) on disease activity index (I) and colon length (J) in mouse DSS model were analyzed. Data represent means ± SD (*n* = 6), **P < 0.01 by 2-way ANOVA and Fisher’s least significant difference (LSD) tests (C, D, and I). Fecal consistency score (K), colon biopsy (L), colonic histology changes (M and N), plasma C-reactive protein (CRP) level (O), and TNF-α in colon tissue (P) of transferrin or hydrocortisone enema-treated idiopathic chronic diarrhea (ICD) rhesus macaques are shown. Data represent means ± SD (*n* = 5), **P < 0.01 by 2-way ANOVA and Fisher’s least significant difference (LSD) tests (K) or unpaired *t* test (N, O, and P). (Q) Graphical abstract of transferrin’s functions in iron transportation, coagulation, and immunosuppression. Microbe infection results in iron withdrawal and promotes transferrin expression. For microbes, the up-regulated transferrin is beneficially used as an immunosuppressor to negatively regulate PRR signaling. For hosts, the up-regulated transferrin promotes immunothrombosis to facilitate the clearance of invading pathogens. Tf, transferrin; HS, histological score.

The pathogenesis of ulcerative colitis (UC) is complex, involving multiple genetic and environmental factors, epithelial barrier damage, gut dysbiosis, and abnormal immunological response [18,51–60]. Human UC is characterized by impaired immunotolerance, which is similar to GF mice [46,47]. UC results from the breakdown in immunotolerance to gut bacteria [47,61]. We next wonder if there is decreased transferrin expression in human UC as found in GF mice. As illustrated in Fig. 7A, decreased transferrin level was found in plasma of UC patients. The average transferrin concentration in the plasma of UC patients (*n* = 20; 10 male and 10 female) was 1.779 mg/ml (SD 0.27), whereas in healthy individuals (*n* = 20; 10 male and 10 female), the average concentration was 2.381 mg/ml (SD 0.37) (Fig. 7A). An obvious decreased level of transferrin was also observed in the colon tissue of UC patients (Fig. 7B).

Two experimental colitis models (dextran sodium sulfate [DSS] and T cell receptor α chain-deficient [TCRαKO] colitis murine models), which result in intestinal dysbiosis and immune imbalance [62–64], and monkeys with spontaneous chronic colitis were used to further investigate the effects of transferrin on chronic dysregulated immune response in intestinal tract. In the DSS colitis model, weight loss in mouse group with transferrin overexpression was lower than that in the control and transferrin knockdown group (Fig. 7C). Compared with the control and transferrin knockdown mice, there was a lower score of disease activity index (Fig. 7D), a longer colon length (Fig. 7E and Fig. S20), and alleviation of inflammation-associated histological changes (Fig. 7F and Fig. S21) in mice of transferrin overexpression. In addition, obvious elevation or reduction in the frequency, number, and proliferation of Foxp3^+^ Tregs in the lymph node of transferrin-overexpression or -knockdown mice was observed, respectively (Fig. 7G and Fig. S22). In the TCRαKO colitis model, alleviation of inflammation-associated histological changes (Fig. 7H and Fig. S23) was observed in transferrin overexpression mice compared with the control and transferrin knockdown mice. Importantly, TC6 blocked transferrin’s improvement on disease activity index (Fig. 7I) and inhibition on colon length shortening (Fig. 7J and Fig. S24) in the colitis model.

Idiopathic chronic diarrhea (ICD) of rhesus macaques leads to dehydration and weight loss affecting as many as 15% of animals in some colonies [65]. This type of chronic colitis resembles UC and may provide clues about the pathogenesis of human inflammatory bowel disease (IBD) [66,67]. By using hydrocortisone (the first line drug to treat UC) as a control, the therapeutic effects of transferrin on ICD were tested. As illustrated in Fig. 7K, the mean fecal consistency score for transferrin enema administration in rhesus macaques showed marked improvement. Alleviation of inflammation-associated colon biopsy scores (Fig. 7L) and colonic histology changes (Fig. 7M and N) was observed in transferrin-treated monkeys. In addition, decreased C-reactive protein (CRP) level in plasma (Fig. 7O) and TNF-α in colon tissue (Fig. 7P) in transferrin treated monkeys were also observed. Generally, transferrin showed better therapeutic effect on ICD than hydrocortisone.

## Discussion

The studies reported here have shown that transferrin, the main iron transporter in serum, is a negative regulator of pattern recognition receptor (PRR) signaling to induce intestinal immunotolerance. Transferrin was up-regulated by the disturbances of iron metabolism as well as by PAMPs including LPS, LTA, and bacterial DNA. Transferrin interacted directly with CD14 to block PRR signaling and consequently to silence host immunity and induce immune tolerance. Here as an endogenous protein, transferrin is firstly reported to regulate the presentation of LPS from CD14 to TLR4. The results show here that, on one hand, microbes possibly beneficially up-regulate host transferrin as a negative regulator of PRRs signaling to counteract immune-mediated iron withdrawal upon infections, but on the other hand, given its function as a clotting regulator in our recent reports [29,68], transferrin up-regulation in host possibly evokes immunothrombosis to defend invading pathogens, demonstrating an important role of transferrin in mediating reciprocal interactions among iron homeostasis, infection, and immunity.

The concentration of transferrin in the plasma is normally 2 to 4 g/l with an iron saturation 15% to 45%. There is a well-described interaction between iron status and immune function, but the link between the abnormalities of immune function associated with disorders of iron homeostasis remains a black box in most cases [69]. We found that bacterial products including LPS, LTA, and bacterial DNA from both pathogenic and probiotic bacteria up-regulated transferrin expression, suggesting that microbe commensalism and/or infections have the ability to up-regulate transferrin expression. As an immune strategy aimed at limiting the microbial iron availability, host immune activation is initiated upon microbe invasion to cause a reduction in plasma iron or iron withdrawal and anemia and iron sequestration (IS) for invading microbes. The up-regulated transferrin by microbes in hosts may act as an iron-mobilizing protein to withhold iron and thus contribute to the IS. Given that iron deficiency up-regulated transferrin as reported [29,30], the host-driven iron withdrawal upon microbial infection likely expands transferrin’s up-regulation induced by microbial products.

Microorganisms stimulate PRRs to initiate a range of host defense mechanisms [70]. In particular, the activation of proinflammatory responses mediated by PRRs is essential for host defense, but excessive inflammation itself is maladaptive [71]. Regulation of PRRs signaling provides one point of control for excessive inflammation. In particular, TLRs play critical roles in immunotolerance and the mechanisms are complex [72]. Gomez-Llorente et al. [72] suggested that TLRs’ negative regulation may be another possible mechanism to induce immunotolerance. Although different types of microbial products (e.g., LPS, LTA, and DNA) studied here are recognized by different TLRs, all of them showed the ability to up-regulate transferrin expression, suggesting that these bacterial products may share a common pathway for the regulation of transferrin expression. We found that NF-κB plays a key role in transferrin up-regulation induced by bacterial products. NF-κB can be activated by members of TLR family that recognize conserved microbial structures (i.e., LPS, LTA, and bacterial DNA) [73] and is a pivotal transcription factor involved in the regulation of a variety of proteins, including transferrin [74]. Here, transferrin up-regulation by bacterial products was inhibited by the specific NF-κB inhibitor CAPE, indicating that transferrin up-regulation promoted by different microbe products is involved in NF-κB activation. This finding reveals that transferrin directly interacted with CD14, which is not only a TLR coreceptor but also a PRR and plays multiple roles in microbial recognition and signaling, to block signal intracellular responses after recognition of a vast array of bacterial products, thus resulting in negative regulation of PRRs signaling and inducing intestinal immunotolerance.

The immunosuppressive function of transferrin was further demonstrated by mouse colitis models induced by DSS and TCRαKO and monkey with spontaneous chronic colitis, which are widely used to study IBD, immune imbalance, and intestinal dysbiosis. The DSS model induces epithelial damage [75], and the TCRαKO colitis murine model develops spontaneous chronic colitis similar to human UC at 16 to 20 weeks of age [76]. Monkey chronic colitis resembles human UC. Transferrin down-regulation impaired host tolerogenic responses by dysregulating DC, Tregs, and Bregs homeostasis in the gut while its overexpression or exogenous administration of transferrin showed protective effects by suppressing the inflammatory response to repair colitis.

The peptide TC6 inhibits transferrin–CD14 interaction but has no effect on LPS’s binding to CD14, and thus does not affect inflammatory responses induced by LPS. TC6 counteracts transferrin’s immunosuppressive functions to block transferrin’s inhibition on LPS-induced inflammatory responses both in vitro and in vivo (Figs. S10G and H, S13, and S19) and to inhibit the protective effects of transferrin on intestinal immune imbalance induced by gut microbial dysbiosis, and consequently diminishes transferrin’s improvement on pathological insults in the colitis models (Fig. S7I and J and Fig. S24), demonstrating that transferrin’s immunosuppressive phenotypes seen in the cell and animal models are mediated by transferrin–CD14 interaction.

In conclusion, our findings revealed that immune activation due to microbe infection results in iron withdrawal, while both infection and iron withdrawal promote host transferrin expression. For microbes, the up-regulated transferrin is beneficially used as an immunosuppressor to negatively regulate PRRs signaling and promote immune tolerance. For hosts, the up-regulated transferrin takes part in iron mobilization to contribute to IS, and also likely promotes immunothrombosis to facilitate the clearance of invading pathogens (Fig. 7Q). Combined with our recent discovery [29,68], transferrin is identified as a multi-tasking plasma protein with functions of iron transportation, coagulation promotion, and immunoregulation. The strong immunosuppressive ability of transferrin by targeting multiple PRRs provides a promising strategy to treat autoimmune diseases, such as IBD.

## Materials and Methods

### Animals and ethics statement

All animal experiments were approved by the Animal Care and Use Committee of the Kunming Institute of Zoology (SMKX-20181115-174) and conformed to the US National Institutes of Health’s *Guide for the Care and Use of Laboratory Animals* (National Academies Press, 8th Edition, 2011). SPF C57BL/6J and GF mice (8 weeks old) were purchased from the Institute of Laboratory Animal Sciences, Chinese Academy of Medical Sciences. TCRα KO mice (#002116, male, 16 weeks old) were purchased from the Jackson Laboratory. All mice were housed under a 12-h light/12-h dark cycle at 24 °C and tested at 10 weeks of age. BsA-treated mice (SPF C57BL/6J) were fed water with ampicillin (1 g/l), streptomycin (1 g/l), metronidazole (0.5 g/ml), and vancomycin (1 g/l) for 3 weeks. Lentivirus for transferrin overexpression (10^7^ transducing units [TU]), retrovirus for transferrin knockdown (10^7^ TU), or their blank viruses (10^7^ TU) were injected into SPF C57BL/6J mice through the tail vein to induce transferrin overexpression or knockdown, and the transferrin concentration was detected periodically until it was successfully overexpressed or knocked down.

### Human plasma and colon tissue specimens from UC patients

The Institutional Review Board of the Kunming Institute of Zoology (KIZ) and the First Affiliated Hospital of Kunming Medical University approved this study (2017L15). All human specimens were collected with the informed consent of patients prior to the study. Plasma and colon tissue samples from UC patients (*n* = 20) and healthy controls (*n* = 20) were collected from the First Affiliated Hospital of Kunming Medical University. In total, 20 subjects with UC showed typical clinical features, including continuous mucosa, diffuse hyperemia, edema, and erosion; blurred or disordered vascular network texture; diffuse erosion and superficial ulcers in lesions; and scattered pseudopolyps, with purulent secretions, white moss, and spontaneous bleeding. Immediately following blood draw (with anticoagulant agent 1.5% EDTA-Na2), plasma was obtained by centrifugation at 3,000 rpm for 20 min at 4 °C and stored at −80 °C after being sub-packed.

### Stimulation assays

Bacterial products including *E. coli* LPS or *B. fragilis* LPS, *S. aureus* LTA, mouse DNA, *E. coli* DNA, *S. aureus* DNA, or *L. monocytogenes* DNA on transferrin expression were determined by Western blotting, ELISA, and qRT-PCR in primary macrophages and BNL CL.2. Detailed information can be found in the Supplementary Materials.

### TLR4 activation assays

THP-1 cells were stimulated by 2 μg/ml LPS mixed with different concentrations of apo-transferrin (0.05, 0.5, and 5 μM) at 37 °C for 2 h. Cells were washed 3 times with phosphate buffer solution (PBS) and incubated with 5 mM of the cross-linking reagent disuccinimidyl suberate (DSS, 21555, Thermo Fisher Scientific, USA) for 30 min at room temperature. After terminating the reaction with 50 mM Tris, cells were washed with ice-cold PBS and lysed in 100 μl of radioimmunoprecipitation assay buffer (R0278, Sigma, USA) with protease inhibitor cocktail. TLR4 dimerization was detected by Western blot analysis as described above. The phosphorylation of TAK1, IKKα, IκBα, NF-κB, and p65 subunits of the myD88-dependent pathway; TBK1 and IRF3 of the myD88-independent pathway; and JNK and p38 of the MAPK signaling pathway in THP-1 cells was also analyzed by Western blotting.

### SPR analysis

BIAcore 2000 (GE, USA) was used to analyze the interaction between transferrin coupled at a CM5 sensor chip (BR100012, GE, USA) and CD14. Detailed information can be found in the Supplementary Materials.

### Murine inflammation model induced by LPS

LPS (750 μg/kg) was intravenously injected into mice (C57BL/6J, male, 8 weeks old) to induce an inflammatory response for 2 h, and effects of transferrin overexpression and transferrin knockdown on LPS-induced inflammatory response were tested. Detailed information can be found in the Supplementary Materials.

### Mice tolerogenic responses assay

Levels of ALDH1A2, CCL22, TGF-β, and IL-10 or frequency, number, and proliferation of CD103^+^CD11b^+^ DCs, Tregs, and Bregs in GF mice and transferrin-knockdown SPF mice were determined by Western blotting or flow cytometry. Detailed information can be found in the Supplementary Materials.

### Colitis animal models

DSS (1.5%)-induced and TCRαKO colitis mice models, and monkeys with spontaneous chronic colitis were used to test the effects of transferrin on dysregulated gut immune response. Detailed information can be found in the Supplementary Materials.

### Statistical analysis

The data obtained from independent experiments were presented as the mean ± SD. All statistical analyses were 2-tailed and with 95% confidence intervals (CIs). The Kolmogorov–Smirnov test was used in the analysis of normal distribution, and data were then analyzed using 1-way ANOVA, or 2-way ANOVA in case of measuring the effects of 2 factors simultaneously, with post-hoc Dunnett or Bonferroni adjustment for *P* values. If only 2 groups were compared, unpaired *t* test was applied. Data were analyzed using Prism 6 (GraphPad Software) and SPSS (SPSS Inc., USA). Differences were considered significant at *P* < 0.05.

## Data Availability

The data that support the findings of this study are available from the corresponding author upon reasonable request.
