# Supplementary material for: Transferrin Is Up-Regulated by Microbes and Acts as a Negative Regulator of Immunity to Induce Intestinal Immunotolerance
Source: Research (Wash D C). 2024 Jan 25;7:0301. doi: 10.34133/research.0301 (PMC10809841; doi:10.34133/research.0301)
Supplement: Supplementary 1 — Figs. S1 to S24 [file research.0301.f1.docx]

**Supplementary Materials**

**Supplementary methods**

**Quantitative real-time polymerase chain reaction (qRT-PCR)**

Cells were harvested. RNA extraction and cDNA reverse transcription were performed using an RNA extraction kit (DP419, Tiangen, China) and reverse transcription kit (A5000, Promega, USA), respectively, as per the manufacturers’ protocols. Transferrin expression was quantified by qRT-PCR (forward primer (5’-3’): GGACGCCATGACTTTGGATG; reverse primer (5’-3’): GCCATGACAGGCACTAGACC for mouse transferrin; and forward primer (5’-3’): CCCTTAACCAATACTTCGGCTAC; reverse primer (5’-3’): GCCAAGTTCTCAAATATAGCTGAG for human transferrin). PCR was performed on a CFX-96 Touch Real-Time Detection System (Bio-Rad, USA).

**Enzyme-linked immunosorbent assay (ELISA)**

Transferrin and cytokines in the samples (cell supernatant or plasma) were measured using mouse transferrin ELISA kit (ab157724, Abcam, USA), mouse TNF-α ELISA kit (DKW12-2720-096, Dakewe Biotech, China), mouse IL-6 ELISA kit (DKW12-2060-096, Dakewe Biotech, China), mouse IL-1β ELISA kit (DKW12-2012-096, Dakewe Biotech, China), mouse IFN-β ELISA kit (SEA222Mu-96T, USCN, China), human transferrin ELISA kit (EK12012, MultiSciences, China), human TNF-α ELISA kit (DKW12-1720-096, Dakewe Biotech, China), human IL-6 ELISA kit (DKW12-1-60-096, Dakewe Biotech, China), human IFN-β ELISA kit (SEA222Hu-96T, USCN, China), and human TGF-β1 ELISA kit (DKW12-1710-096, Dakewe Biotech, China), respectively, according to the manufacturers’ instructions.

**Western blotting**

Total proteins were extracted by RIPA Buffer (R0278, Sigma-Aldrich, USA) containing protease inhibitors (HY-K0010, MedChem Express, USA) and phosphatase inhibitors (HY-K0022, MedChem Express, USA). Samples were separated by 12% sodium dodecyl sulfate-polyacrylamide gel electrophoresis (SDS-PAGE) and further transferred to polyvinylidene difluoride (PVDF) membranes. The membranes were blocked with 5% bovine serum albumin (BSA) dissolved in [TBST](http://www.thelabrat.com/protocols/TBST.shtml) buffer (2.42 g/L Tris base, 8 g/L NaCl, 0.1% Tween-20 (v/v), pH 7.6) for 2 h at room temperature. After washing three times with TBST buffer, the PVDF membranes were incubated overnight in primary antibody at 4°C, followed by three washing in TBST buffer and further incubation in secondary antibody at room temperature for another 1 h. Subsequently, the membranes were washed again with TBST buffer and then developed with an enhanced chemiluminescence kit (PA112, Tiangen, China) using an ImageQuant LAS 4000 mini (GE Healthcare, USA). The primary antibodies included anti-phospho-transforming growth factor-β (TGF-β)-activating kinase 1 (TAK1) (1:1 000 dilution, AF4379-100ul, Affinity Biosciences, USA), anti-total-TAK1 (1:1 000 dilution, AF4679-50ul, Affinity Biosciences, USA), anti-phospho-inhibitory subunit of nuclear factor κB (NF-κB) (IκB) kinase α (IKKα) (1:1 000 dilution, C84E11, Cell Signaling Technology, USA), anti-total-IKKα (1:1 000 dilution, ab32041-40ul, Abcam, USA), anti-phospho-IκBα (1:1 000 dilution, AF2002-100, Affinity Biosciences, USA), anti-total-IκBα (1:2 000 dilution, 4814T, Cell Signaling Technology, USA), anti-phospho-NF-κB p65 (1:1 000 dilution, AF2006-100, Affinity Biosciences, USA), anti-total-NF-κB p65 (1:1 000 dilution, 6956T, Cell Signaling Technology, USA), anti-phospho-TRAF-associated NF-κB activator (TANK)-binding kinase 1 (TBK1) (1:1 000 dilution, AF8190-50 μl, Affinity Biosciences, USA), anti-total-TBK1 (1:1 000 dilution, DF7026-50, Affinity Biosciences, USA), anti-phospho-interferon regulatory factor 3 (IRF3) (1:1 000 dilution, 29047S, Cell Signaling Technology, USA), anti-total-IRF3 (1:1 000 dilution, ab50772, Abcam, USA), anti-phospho-c-JUN N-terminal kinase (JNK) (1:1 000 dilution, 9255S, Cell Signaling Technology, USA), anti-phospho-p38 (1:1 000 dilution, 4511S, Cell Signaling Technology, USA), anti-ALDH1A2 (1:1 000, DF4422, Affinity Biosciences, USA), anti-CCL22 (1:1 000, DF7781, Affinity Biosciences, USA), anti-TGF-β1(1:1 000,50698-T48, Sino Biological, China), anti-IL-10 (1:1 000, GTX130513, GeneTex, USA), anti-toll-like receptor 4 (TLR4) (1:1 000, AF7017, Affinity Biosciences, China), anti-transferrin (1:5 000, ab82411, Abcam, USA), anti-GAPDH (1:3 000, T0004-50, Affinity Biosciences, USA) and anti-β-actin (1:3 000, T0022-50, Affinity Biosciences, USA).

**Bacterial and mouse DNA preparation**

Bacterial isolates of gram-negative (*Escherichia coli* ATCC 9637) and gram-positive bacteria (*Staphylococcus aureus* ATCC 25923) and *Listeria monocytogenes* ATCC 19115) were used for DNA extraction. Briefly, *E. coli* and *S. aureus* were cultured in Luria-Bertani (LB) medium (CAI-LBP03-500GM, Caisson Labs, USA). *L. monocytogenes* was cultured in brain heart infusion (BHI) broth (Diagnostics Pasteur, Marnes la Coquette, France). Bacterial cultures at the logarithmic phase were collected, and total bacterial DNA was extracted using a bacterial DNA extraction kit (DP302-02, Tiangen, China) as per the manufacturer’s protocols. Mouse DNA was extracted from the liver using an extraction kit (DP341-01, Tiangen, China) as per the manufacturer’s instructions. Enterotoxin in DNA samples was tested using a commercial Enterotoxin ELISA kit (abx054396, abbexa, UK).

**Cell culture**

The human monocytic cell line (THP-1) was obtained from the Conservation Genetics CAS Kunming Cell Bank (Kunming, China) and maintained in RPMI1640 medium (Gibco Laboratories, USA) containing 10% fetal bovine serum (FBS; MRC, Australia) at 37°C and in the presence of 5% CO_2_. The mouse normal embryonic liver cell line (BNLCL.2) was also obtained from the Cell Bank and maintained in Dulbecco’s modiﬁed Eagle’s medium (DMEM, Gibco Laboratories, USA) containing 10% FBS at 37°C and under 5% CO_2_. The medium was changed every 3 d and experiments were conducted between the third and eighth passages.

**Isolation of human peripheral blood mononuclear cells (PBMCs) and** **polymorphonuclear neutrophils (PMNs)**

Healthy human peripheral blood treated with 1.5% EDTA-Na_2_ anticoagulant agent was collected from the Kunming Blood Center, Yunnan Province, China. PBMCs and PMNs were isolated by using Polymorphprep (AS1114683, Axis-Shield, Norway) as per the manufacturer’s protocols. In brief, 5 ml of anticoagulated peripheral blood was carefully layered over 5 ml of Polymorphprep, and the samples were then centrifuged at 500 g for 30 min in a swing-out rotor at 22°C. After the centrifugation, two leukocyte bands were visible. The top band at the plasma/Polymorphprep consisted of mononuclear cells and the lower band consisted of polymorphonuclear cells. The two bands were harvested using a pipette, and the fractions of the two bands were diluted with one volume of RPMI 1640 medium. The cell suspension was transferred to a 3-ml tube and centrifuged three times at 500 g for 30 min at 22°C. The isolated PBMCs and PMNs were maintained in RPMI1640 medium containing 10% FBS at 37 °C and in the presence of 5% CO_2_. Primary macrophage was isolated from PBMCs using macrophage differentiation kit (CDK012, CellXVivo, USA) as per the manufacturer’s protocols.

**Isolation of dendritic cells from bone marrow**

Femurs and tibiae of C57BL/6J mice (male, 8 weeks old) were removed and purified from the surrounding muscle tissue. The bones were left in 70% ethanol for 5 min for disinfection and washed twice with phosphate buffer solution (PBS). Both ends of the bones were cut with scissors. The marrow was flushed out with complete medium (CM, RPMI 1640 supplemented with 10% FBS, 2 mM L-glutamine, 1% of nonessential amino acids, 100 U/ml penicillin, and 100 μg/ml streptomycin) using a syringe with a 0.45-mm needle. Clusters within the marrow suspension were disassociated by pipetting and filtrated through a 70-μm sterilized cell strainer. Red blood cells in the suspension were lysed with Ammonium-Chloride-Potassium (ACK) lysing buffer (R1010-100, Solarbio, China). Bone marrow cells (BMs) were seeded at a concentration of 1×l0^6^ cells/ml in RPMI 1640 medium supplemented with 20 ng/ml GM-CSF (415-ML-050, R&D Systems, USA). After 7 d of culture, the bone marrow dendritic cells (BMDCs) were harvested, counted, and used for the experiments.

**Stimulation assays**

Primary macrophages and BNLCL.2 were first seeded into 24-well plates at a density of 2.5 × 10^5^ cells per well for 4 h. In order to test the effects of bacterial products including LPS, lipoteichoic acid (LTA) and bacterial DNA on transferrin expression, the cells were seeded into 24-well plates at 4 × 10^5^ cells/well and maintained for 12 h in the corresponding medium. Different concentrations (0.08, 0.4, and 2 μg/ml) of *E. coli* LPS (L2630-10MG, Sigma, USA) or *B. fragilis* LPS as extracted above, *S. aureus* LTA (0.4, 2, and 10 μg/ml; L2525-5mg, Sigma, USA), mouse DNA (0.4, 2, and 10 μg/ml), *E. coli* DNA (0.4, 2, and 10 μg/ml), *S. aureus* DNA (0.4, 2, and 10 μg/ml), or *L. monocytogenes* DNA (0.4, 2, and 10 μg/ml) were then added and incubated with the cells for 24 h at 37 °C after replacing with [serum-free](javascript:void(0);) [medium](javascript:void(0);). The cells were pretreated for 30 min by the specific NF-κB inhibitor caffeic acid phenethyl ester (CAPE, HY-N0274, MCE, USA) (20 μM) to test the inhibitor’s effects on transferrin expression induced by LPS (2 μg/ml), LTA (10 μg/ml), *E. coli* DNA (10 μg/ml), *S. aureus* DNA (10 μg/ml), or *L. monocytogenes* DNA(10 μg/ml). The transferrin levels in cells were analyzed by Western blotting using anti-transferrin antibody (1:5 000 dilution, ab82411, Abcam, USA) as described above, with β-actin used as the loading control. The cell supernatant transferrin level was measured by using the ELISA kit described above. Total RNA in cells was extracted using an RNA Extraction Kit to test the expression of transferrin by qRT-PCR according to the method described above.

In order to test effects of transferrin or its antibody on inflammatory cytokines secretion, THP-1 cells, HUVECs, PBMCs, PMNs, or BMDCs were seeded into 24-well plates at 4 × 10^5^ cells/well and maintained for 4 h and then incubated with anti-transferrin receptor (TfR) antibody (10 μg/ml, ab1086, Abcam, USA) for 30 min. Different concentrations (0.05, 0.5, and 5 μM) of apo-transferrin (T4382, Sigma, USA) or holo-transferrin (T4132, Sigma, USA) were added and incubated with cells for 10 min before LPS (2 μg/ml) addition to stimulate cells for 12 h**.** Supernatant levels of TNF-α, IL-6, IFN-β, and TGF-β were measured by using ELISA kits.

**NF-κB reporter assays**

Primary macrophages were seeded into a 96-well plate for 24 h. The cells were then transfected with pNL3.2.NF-κB-RE [NlucP/NF-κB-RE/Hygro] plasmids (N111A, Promega, USA). The culture medium was replaced with fresh medium containing different concentrations of transferrin for 30 min, followed by the treatment with 2 μg/ml LPS for 6 h. Luciferase activity was determined by using the Nano-Glo® Luciferase Assay System (N1110, Promega, USA), and relative luciferase fold change was calculated.

**Surface plasmon resonance (SPR) analysis**

BIAcore 2000 (GE, USA) was used to analyze the interaction between transferrin and CD14 by using albumin (NPB028Hu01, USCN, China) as the control. Transferrin or human serum albumin was first diluted (20 μg/ml) with 200 μl of sodium acetate (10 mM, pH 5), with the transferrin solution then flowed across the activated surface by NHS (N-hydroxysuccinimide) and EDC (1-ethyl-3-[3-dimethylaminopropyl] carbodiimide hydrochloride) of the CM5 sensor chip at a flow rate of 5 μl/min to couple with a CM5 sensor chip (BR100012, GE, USA) to a 500-target response value (RU). The remaining activated sites on the CM5 sensor chip were blocked by 75 μl of ethanolamine (1 M, pH 8.5). Different concentrations of CD14 (125, 250, 500, and 1000 nM; SEA518Mu-48T, Uscn, China) in Tris-HCl buffer (20 mM, pH 7.4) were applied to analyze interactions with transferrin or albumin on the surface of the CM5 sensor chip at a flow rate of 20 μl/min. The purity of all purchased proteins was greater than 98%. The equilibrium dissociation constant (*KD*) for binding, as well as the association (*Ka*) and dissociation (*Kd*) rate constants, were determined by the BIA evaluation program (GE, USA). The LPS binding region of the CD14 N-terminus (elddedfrcvcnfsepqpdwseafqcvsaveveihaggln) deduced from the human CD14 sequence (GenBank number (NP_000582.1) and its scrambled peptide (vdlsleeenveprdvesqfncesfwacdhggcqfapvadi) were synthesized by GL Biochem (Shanghai, China) with a purity >98% and also applied to analyze their interactions with transferrin by SPR. In addition, SPR was also used to determine if there was an interaction between LPS (L2630-10MG, Sigma, USA) and transferrin. All of commercial acquired transferrin in this study has no LPS contamination determined by using ToxinSensor kit (LF350, Genscript, China).

The effects of transferrin on LPS-CD14 interaction were also investigated by SPR. LPS antibody (10 μg/ml, MAB526Ge22-100ul, USCN, China) was dissolved in sodium acetate (10 mM, pH 5.5) and coupled with the CM5 sensor chip as mentioned above. LPS (1 mg/ml) was then flowed across the CM5 sensor chip to combine with the immobilized LPS antibody. CD14 (1 μM) mixed with different concentrations of transferrin (0.05, 0.5, and 5 μM) was applied to analyze the blockage of transferrin on LPS -CD14 interaction at a flow rate of 20 μl/min. Native PAGE was used to analyze the interactions between transferrin and CD14 as per the protocols described previously (*1*).

**Immunoprecipitation**

Cell lysis sample of primary macrophages was acquired, and then anti-transferrin antibody (5μg, ab82411, Abcam, USA) was added and incubated for 16 h at 4°C in 30 μl of Tris-HCl buffer (25 mM, pH 7.4). Protein A agarose (20 μl, P2006, Beyotime, China) was then added and incubated for 3 h at 4°C. After the centrifugation at 2 500 rpm for 5 min at 4°C, loading buffer (10 μl, 4×CW0027A, CWBIO, China) was added, followed by boiling for 10 min to obtain the coupled proteins. All proteins were subjected to 12% SDS-PAGE separation and polyclonal antibodies against CD14 (DF13278, Affinity, USA) was used to identify corresponding protein, respectively.

**Protein-protein docking**

To model the transferrin-CD14 complex, we used the known structure of transferrin and CD14 for protein docking. The crystal structure of transferrin (PDB ID: 3V83) (DOI: 10.1038/nature10823) was docked to the structure of CD14 (PDB ID: 4GLP) (DOI: 10.1002/cbic.201402620) by ZDOCK. In predicting protein-protein complexes, ZDOCK considers shape complementarity, electrostatics, and desolvation free energy (https://doi.org/10.1002/prot.10389). Protein-protein docking was guided by transferrin activity and SPR experimental data (Figure 2A), where residues that disrupted binding were forced to be included in the interface and residues that did not affect binding were forced not to be included. About 2 000 structure complexes were generated and ranked according to the ZRANK scoring function. The best ZDOCK pose between the two conformations was used as a representative of transferrin-CD14 interaction.

**Recombinant proteins expression**

The prokaryotic expression vector was constructed by inserting the DNA sequence encoding mature transferrin (GenBank: AAA61140.1, 679 amino acids) or transferrin mutant (R663A and K664A) between the KpnI and XhoI sites of the pSmart-I vector. The DNA encoding CD14 (NP_000582.1, 356 amino acids) or CD14 mutant (D44A, S46A and Q50A) was inserted between the BamHI and XhoI sites of the pSmart-I vector. The vectors were transformed into *E. coli* Rosetta (DE3), which was induced by 0.8 mM isopropyl β-d-thiogalactoside (IPTG) for 6 h in a 110-rpm shaker at 28 °C. After the induction, *E. coli* cells were collected by centrifugation at 12 000 rpm for 15 min at 4 °C and resuspended in binding buffer (20 mM Tris-HCl, 500 mM NaCl, 5 mM imidazole, pH 7.4). The cells were then homogenized using an Ultrasonic Cell Disruption System (XINYI-IID, XinYi, China). The supernatant was collected by centrifugation at 12 000 rpm for 2 h at 4 °C. The Ni^2+^ affinity chromatography column was equilibrated in advance with binding buffer. The collected supernatant containing fusion protein was subsequently loaded on the Ni^2+^ affinity chromatography column at a flow rate of 0.7 ml/min. The bound fusion proteins were eluted with five column volumes of elution buffer (20 mM Tris-HCl, 250 mM NaCl, 500 mM imidazole, pH 7.4). The eluted fraction was resuspended in 20 mM Tris-HCl, pH 7.8, and the salt was removed by using an ultrafiltration device (UFC500324, Millipore, USA).

For the release of recombinant proteins, small ubiquitin-Like modifier (SUMO) protease (1 unit, 12588-018, Life Technologies, USA) was added to the reaction buffer (50 mM Tris-HCl, 0.2% Igepal, 1 mM dithiothreitol (DTT), pH 8.0) and maintained at 4 °C for 16 h. The reaction buffer was loaded on the Ni^2+^ affinity chromatography column again to remove SUMO protease and fusion tags.

**Fluorescent labeling of proteins**

Transferrin or CD14 (20 mg/ml, dissolved by carbonate-bicarbonate solution (CBS, 1.59 g/L Na_2_CO_3_, 2.93 g/L NaHCO_3_, pH 9.6)) was put in a tube in a dark room. FITC buffer (5 mg/ml, dissolved with CBS; HY-66019, MCE, USA) was added into transferrin or CD14 buffer drop by drop, and then the mixture was incubated for 24 h at room temperature. Sephadex G-25 column (30 × 2 cm, GE, USA) was used to purify the FITC-labeled transferrin (FITC-Tf) or CD14 (FITC-sCD14), and PBS was used as the elution buffer. The purified FITC-labeled transferrin or CD14 was freeze-dried for further use.

**Confocal microscopy**

An immunofluorescent assay was used to analyze the nuclear translocation of the phospho-NF-κB p65 and phospho-IRF3 subunits in THP-1 cells. Cells were incubated with 2 μg/ml LPS mixed with different concentrations of apo-transferrin (0.05, 0.5, and 5 μM) in the corresponding medium described above at 37 °C for 30 min. After the fixation for 15 min in 4% paraformaldehyde in PBS, cells were blocked for 1 h at room temperature with 1% BSA containing 0.2% Triton X-100. After washing with PBS, cells were incubated with anti-phospho-NF-κB p65 (1:200 dilution, AF2006-100, Affinity, USA) or anti-phospho-IRF3 antibody (1:200 dilution, 29047S, CST, USA) for 1 h at 37 °C. After washing three times with PBS to remove excess primary antibodies, cells were incubated with a Cy3-labeled anti-rabbit IgG (H+L) secondary antibody (1:200 dilution, 072-01-15-06, KPL, USA) for 1 h at 37 °C, and washed again with PBS to remove excess secondary antibodies. The cells were then stained with Prolong Gold Antifade 4, 6-diamidino-2-phenylindole (DAPI, P36941, Life Technologies, USA) and imaged with a confocal microscope (FluoView™ 1000, Olympus, USA).

To colocalize transferrin-CD14 complex on the membrane surface of THP-1 cells, FITC-labeled transferrin (0.5 μM) with or without anti-transferrin antibody (10 μg/ml) or control IgG (10 μg/ml) was first incubated with cells for 5 min in the corresponding medium described above, and then LPS (2 μg/ml) was incubated with cells for 30 min. After washing with PBS, cells were fixed for 15 min with 4% paraformaldehyde in PBS, blocked for 1 h at room temperature with 1% BSA, and then incubated with the antibody against CD14 (1:200 dilution, 10073-RP0, Sino Biological, China) for 1 h at 37 °C. The following treatments to remove excess primary and second antibodies, stain and image cells were as above. The similar confocal microscope procedure was performed to observe TLR4-CD14 complex on the membrane surface of THP-1 cells by using FITC-labeled sCD14 (0.5 μM) mixed with different concentrations of transferrin (0.05, 0.5, and 5 μM).

**Generation of lentiviral or retroviral vectors and virus package for transferrin overexpression or knockdown**

Transferrin overexpression or knockdown vectors were constructed. HEK 293T cells (Conservation Genetics CAS Kunming Cell Bank, China) and EcoPack™ 2–293 cells (Clontech, USA) were used to package lentiviruses and retroviruses, respectively, as per our previous report(*1*).

**Murine inflammation model induced by LPS**

C57BL/6J mice (male, 8 weeks old) were used. The lentivirus for transferrin overexpression (10^7^ transducing units (TU)), retrovirus for transferrin knockdown (10^7^ TU), or their blank viruses (10^7^ TU) was injected into C57BL/6J mice through the tail vein to induce transferrin overexpression or knockdown, with transferrin concentration detected periodically. LPS (750 μg/kg) was injected into the tail vein of all mouse groups to induce an [inflammatory](javascript:void(0);) [response](javascript:void(0);) for 2 h. In the transferrin-treated group, LPS injection was performed after mouse transferrin (purity greater than 98%, T0523, Sigma, USA) administration through the tail vein for 20 min. Plasma levels of TNF-α, IL-6, IL-1β, IFN-β, alanine transaminase (ALT) and aspartate aminotransferase (AST) were measured by using ELISA kits. Iron level of plasma and liver tissue of all mice groups was determined by using corresponding kit (ab239715, Abcam, USA).

Mice were injected intravenously with LPS (15 mg/kg) or [*E.*](javascript:void(0);) [*coli*](javascript:void(0);) (10^9^ CFU/kg). Lethality was monitored daily for 7 days and the percentage of dead animals (dead/total) was calculated.

**Histological evaluation**

The harvested liver tissues of all mouse groups were fixed in 4% buffered formalin overnight and dehydrated in 40% sucrose for 2 h. To assess morphological changes, frozen slices (8 μm) were then prepared using a freezing microtome (Cm3050, Leica, Germany) and stained with hematoxylin and eosin using a commercially available kit (G1120-100, Solarbio, China) as per the manufacturer’s protocols. Histology was quantified as previous described(*2*). Additional slices were processed for immunostaining of apoptotic nuclei using an apoptosis detection kit (40307ES20, Yeasen, China) following the manufacturer’s protocols and imaged by an Olympus FluoView 1000 confocal microscope.

**Segmentation of** **intestines and gut-draining lymph nodes (gLN)**

Segmentation of intestines and gut-draining lymph nodes was carried out as described previously(*3*). The duodenum (D), jejunum (J), ileum (I), caecal colon (C1), and ascending colon (C2) were isolated and all peritoneal gLNs (D-gLNs, J-gLNs, I-gLNs, C1-gLNs, and C2-gLNs) from mice were isolated. For segmentation of the small intestine, the upper 25% was taken as the duodenum, the next 50% as the jejunum, and the last 25% as the ileum. Mesenteric lymph node draining intestinal segments were identified anatomically by following the lymphatic vessels connecting the colon, caecum, ileum, and jejunum to their lymph nodes. Duodenal lymph nodes were revealed by gavaging with 100 μl of olive oil and determining the most stomach-proximal lymph nodes surrounded by chyle, indicative of duodenal drainage, 1 h after gavage.

**Isolation of lymphocytes and antigen-presenting cells (APCs) from lymph nodes**

Lymph nodes were chopped into cold Hank's balanced salt solution (HBSS) (SH30030.02B, Gibco, USA) supplemented with 1 mg/ml collagenase D (11088866001, Roche, USA) and incubated at 37°C in 5% CO_2_ for 25 min. Collagenase was quenched on ice by the addition of 10% FBS. Single cell suspensions were extracted by pipetting the digest. Erythrocytes were lysed by incubation in erythrocyte lysis buffer for 5 min at room temperature. Cell suspensions were passed through a 100-μm mesh and stained for flow cytometry.

**Isolation of** **lymphocytes and APCs from gut tissues**

Lymphocytes and APCs were isolated as described previously(*3*). Intestines were cut longitudinally and washed with PBS to remove the digest. Tissues were cut into 1-cm pieces and incubated in PBS with 1 μM DTT for 10 min to remove mucus. The epithelium was removed by centrifugation at 230 rpm after the incubation in 20 ml of HBSS, 2% FBS, and 30 mM EDTA for 10 min at 37°C. After washing with PBS, tissues were finely chopped and digested in 5 ml of RPMI1640 with 2% FBS, 200 μg/ml DNaseI (10104159001, Roche, USA), and 2 mg/ml collagenase 8 (C2139, Sigma, USA) per gut segment for 45 min at 37 °C. Digests were passed through a sieve and centrifuged. Cell pellets were resuspended in 30% Percoll (MB3011, Meilunbio, China) complemented with RPMI1640, 2% FBS,and passed through a 100-μm mesh and separated by centrifugation in a discontinuous Percoll gradient (80%/30%) at 1 000 g for 25 min at room temperature. APCs and lymphocytes were isolated from the interphase, washed, and stained for flow cytometry.

**Flow cytometry**

Fluorescent-dye-conjugated antibodies were purchased from BD Biosciences (USA) (anti-CD45.2,560693; anti-CD4, 553046; anti-CD11c, 550261), eBioscience (USA) (anti-RORγT, 25-6981-82; anti-Foxp3, 12-5773-82; anti-CD1d, 17-0011-82; anti-CD5, 12-0051-82; anti-CD103, 11-1031-81; anti-MHCII, 25-5321-82), and Cell Signaling Technology (USA) (anti-CD19, 54508; anti-CD11b, 85601s). For flow cytometry of dendritic cells (DCs) (anti-CD45.2, anti-MHCII, anti-CD11c anti-CD103, and anti-CD11b), regulatory T cells (Tregs) (anti-CD45.2, anti-CD4, anti-Foxp3, and anti-RORγT), and regulatory B cells (Bregs) (anti-CD19, anti-CD1d, and anti-CD5), the isolated cells described above were surface stained using a subset of antibodies. For the detection of Foxp3 and RORγT, cells were fixed and stained using a Foxp3-staining kit (00-5523-00, eBioscience, USA) according to the manufacturer’s instructions. Dead cells were excluded by propidium iodide (PI) staining. Staining was carried out as described(*4*) and analyzed on a BD LSRFortessa Cell Analyzer (BD Biosciences, USA).

**Colitis animal models**

For dextran sulphate sodium salt (DSS) treatment, C57BL6/J mice were treated with 1.5% DSS (MP Biochemicals, Canada) in drinking water for 7 d and then received regular drinking water for another 4 d. The lentiviruses or retroviruses for transferrin overexpression or knockdown were injected two weeks before DSS treatment. The mice were monitored and scored daily for body weight, presence of diarrhea, stool consistency, and bloody stools. The histological grade of colonic inflammation, colon length, and scoring of disease activity index were also checked in accordance with previously reported standards(*5*). For the T cell receptor α chain-deficient (TCRαKO) model, mice were injected with the lentiviruses or retroviruses for transferrin overexpression or knockdown at 16 weeks of age, and the severity of intestinal inflammation was evaluated by histological analysis at 20 weeks as per previously described criteria(*6*).

**Peptide synthesis**

TC6 (TTPEPC) deduced from the human CD14 sequence (GenBank number (NP_000582.1) was synthesized by GL Biochem (Shanghai, China) with a purity >98% and applied to analyze the interactions with LPS, CD14 or transferrin by SPR. The effect of TC6 on LPS- or transferrin-CD14 interaction were also investigated by SPR. ELISA was used to prove the effect of TC6 on inhibition of cytokines release elicited by transferrin in primary macrophages or mice plasma as the method described above. The effect of TC6 on frequency, number, and proliferation of Tregs in gut lymph nodes was also verified by flow cytometry as the method described above. Effect of TC6 treatment on disease activity index and colon length in mouse DSS colitis model were also analyzed as the method described above.

**Idiopathic chronic diarrhea (ICD) model of rhesus macaques**

Rhesus macaques (~ 8 kg, n = 5) diagnosed with chronic diarrhea were enrolled in this study. These rhesus macaques were housed at the Kunming Primate Research Center of the Chinese Academy of Sciences (KPRC). Transferrin (28 mg) or hydrocortisone (28 mg) was dissolved with 50 ml of saline, and enema administration was performed once a day for 28 days. Fecal consistency was first evaluated prior to cage cleaning every day. The fecal consistency was described as normal, semisolid, liquid, or a combination thereof and was divided into 7-point scoring as previous described(*7*). For the colonoscopy, rhesus macaques were sedated and placed in operating table. The scope (GIF-XQ240, Olympus, Japan) was lubricated, and inserted into the rectum approximately 20 cm proximal to the rectum. Totally 8 colon tissue samples were collected, and 4 biopsy samples were placed into a 4% buffered formalin for histological evaluation. To assess morphological changes, frozen slices (8 μm) were then prepared using a freezing microtome (Cm3050, Leica, Germany) and stained with hematoxylin and eosin using a commercially available kit (G1120-100, Solarbio, China) as per the manufacturer’s protocols. The remaining samples were placed in RNAlater (DP408, Tiangen, China) for analysis of TNF-α mRNA. Plasma level of c-reactive protein (CRP) was measured using the ELISA kit (CSB-E10035Mo, Cusabio, China).

**Supplementary Figure and Figure legends**

**
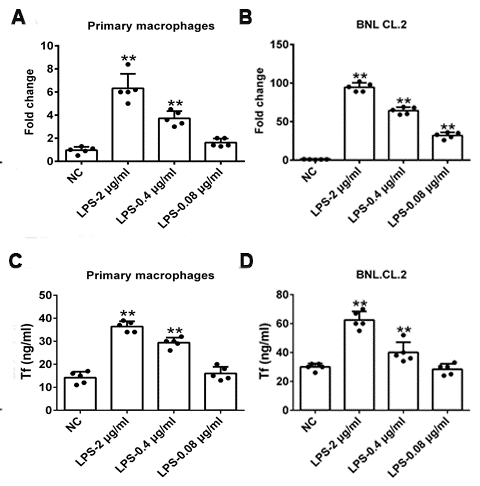
**

**Figure S1. Transferrin is up-regulated by lipopolysaccharide.** *Transferrin* RNA expression in primary macrophages **(A)** and BNL CL.2 cells **(B)** induced by lipopolysaccharide (LPS) from *E. coli* determined by qRT-PCR. Mean ΔCt value of negative control (NC) in **A** or **B** was 4.951 or 4.135, respectively. Transferrin protein levels in primary macrophages **(C)** and BNL CL.2 cells **(D)** induced by LPS analyzed by ELISA. Data represent means ± SD of five independent experiments, **p*< 0.05, ***p*< 0.01 by one-way ANOVA with Dunnett’s *post-hoc* test. Tf: transferrin

**
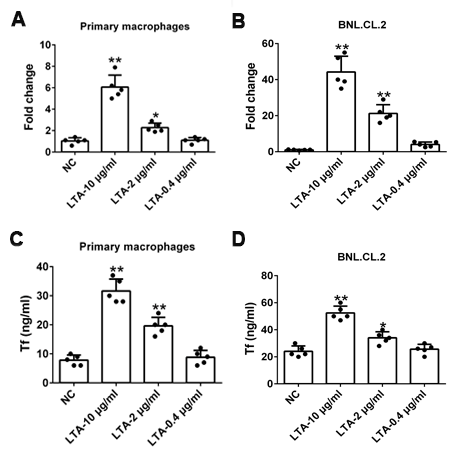
**

**Figure S2. Transferrin is up-regulated by lipoteichoic acid.** *Transferrin* RNA expression in primary macrophages **(A)** and BNL CL.2 cells **(B)**, induced by lipoteichoic acid (LTA) determined by qRT-PCR. Transferrin protein levels in primary macrophages **(C)** and BNL CL.2 cells **(D)** induced by LTA analyzed by ELISA. Data represent means ± SD of five independent experiments, **p*< 0.05, ***p*< 0.01 by one-way ANOVA with Dunnett’s *post-hoc* test. Tf: transferrin.


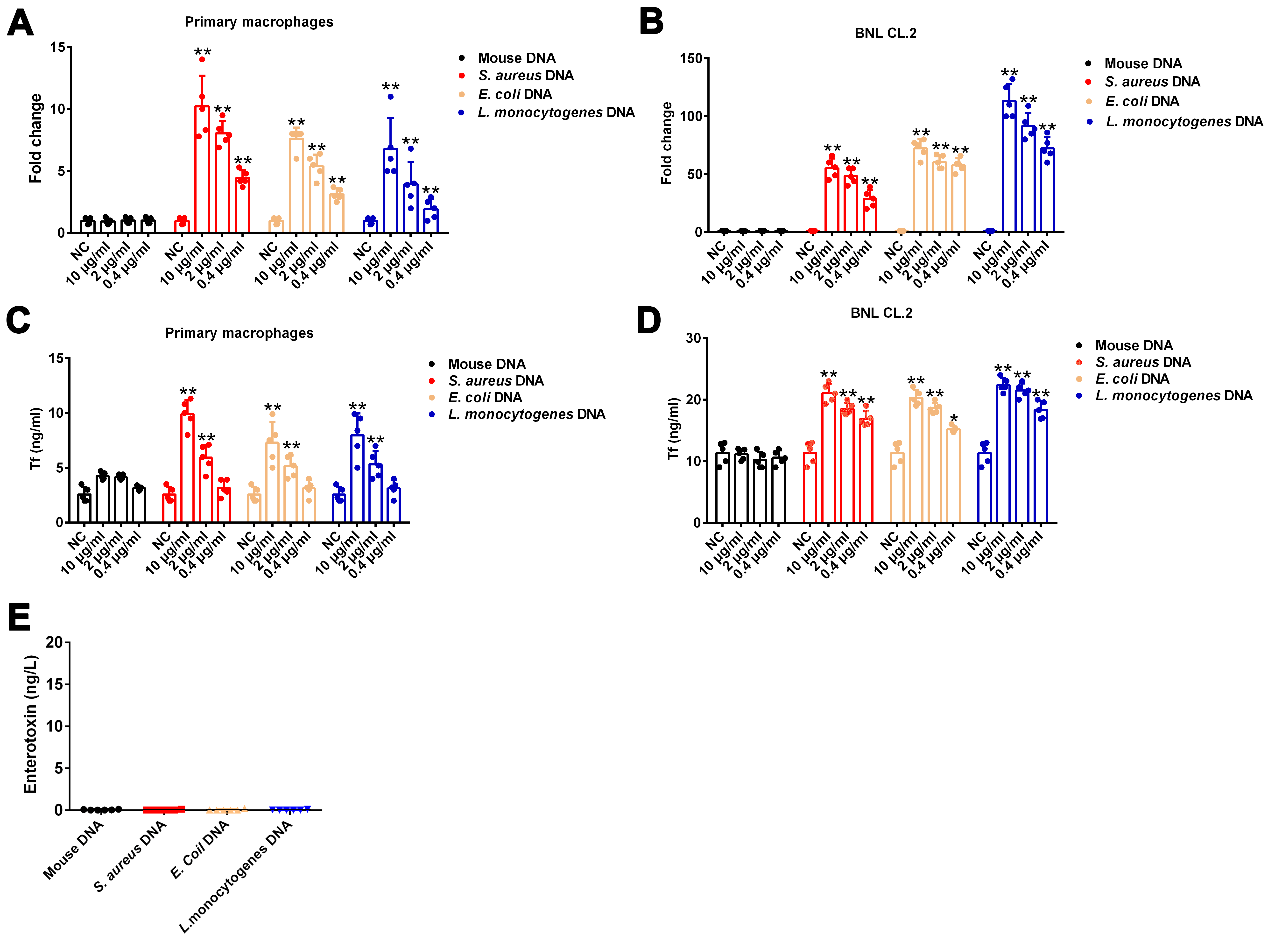


**Figure S3. Transferrin is up-regulated by bacterial DNA.** *Transferrin* RNA expression in primary macrophages **(A)** and BNL CL.2 cells **(B)** induced by DNA from mouse, *E. coli*, *S. aureus*, or *L. monocytogenes* determined by qRT-PCR. Transferrin protein levels in primary macrophages **(C)** and BNL CL.2 cells **(D)** induced by the DNAs analyzed by ELISA. **(E)** Enterotoxin in DNA samples was determined by using Enterotoxin ELISA kit. Data represent means ± SD of five or six independent experiments, **p*< 0.05, ***p*< 0.01 by one-way ANOVA with Dunnett’s *post-hoc* test. Tf: transferrin.


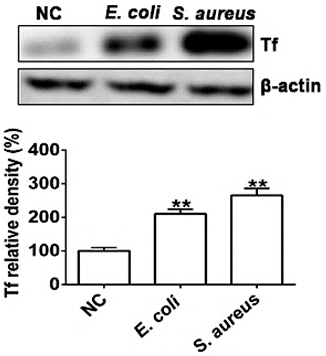


**Figure S4. Transferrin is up-regulated by bacterial pathogens.** Effects of [*Escherichia*](javascript:void(0);) [*coli*](javascript:void(0);) (*E. coli*, 10^3^/ml) *or* [*Staphylococcus*](javascript:void(0);) [*aureus*](javascript:void(0);) (*S. aureus*, 10^3^/ml) treatment for 24 h on transferrin expression in primary macrophages were analyzed by Western blotting. Corresponding quantification are shown on bottom. Data represent means ± SD of five independent experiments, **p* < 0.05, ***p* < 0.01 by by unpaired *t*-test.

**
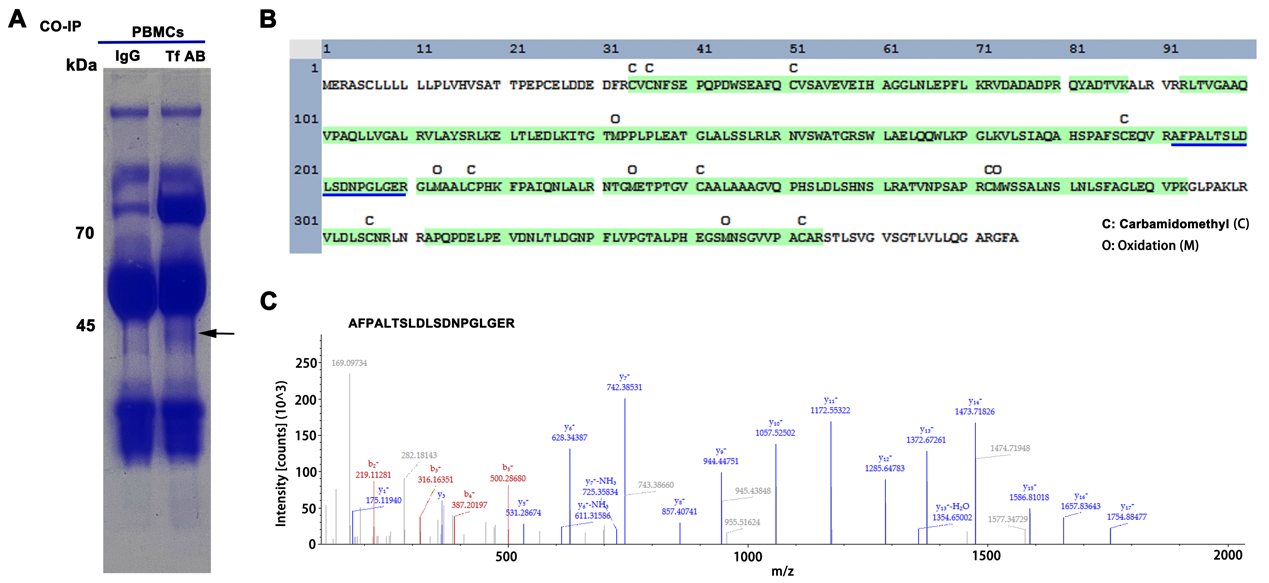
**

**Figure S5. Screening of transferrin targets by mass spectrometry-based co-immunoprecipitation analysis. (A)** 12% SDS-PAGE of transferrin-target proteins in human PBMCs. **(B)** One binding protein was identified as CD14 by LC-MS. Resulting sequence fragments are marked in green shadow. (**C)** Peptide underlined in ‘B’ was further identified by secondary MS analysis. IgG: immunoglobulin G; Tf AB: transferrin antibody.


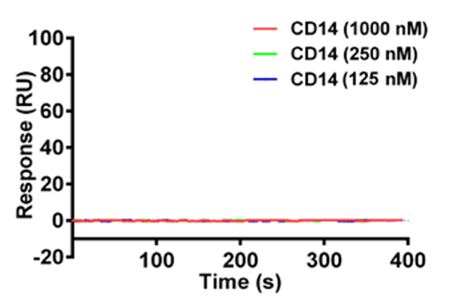


**Figure S6. SPR analysis of human serum albumin-CD14 interactions.** Human serum albumin showed no interaction with CD14.

**
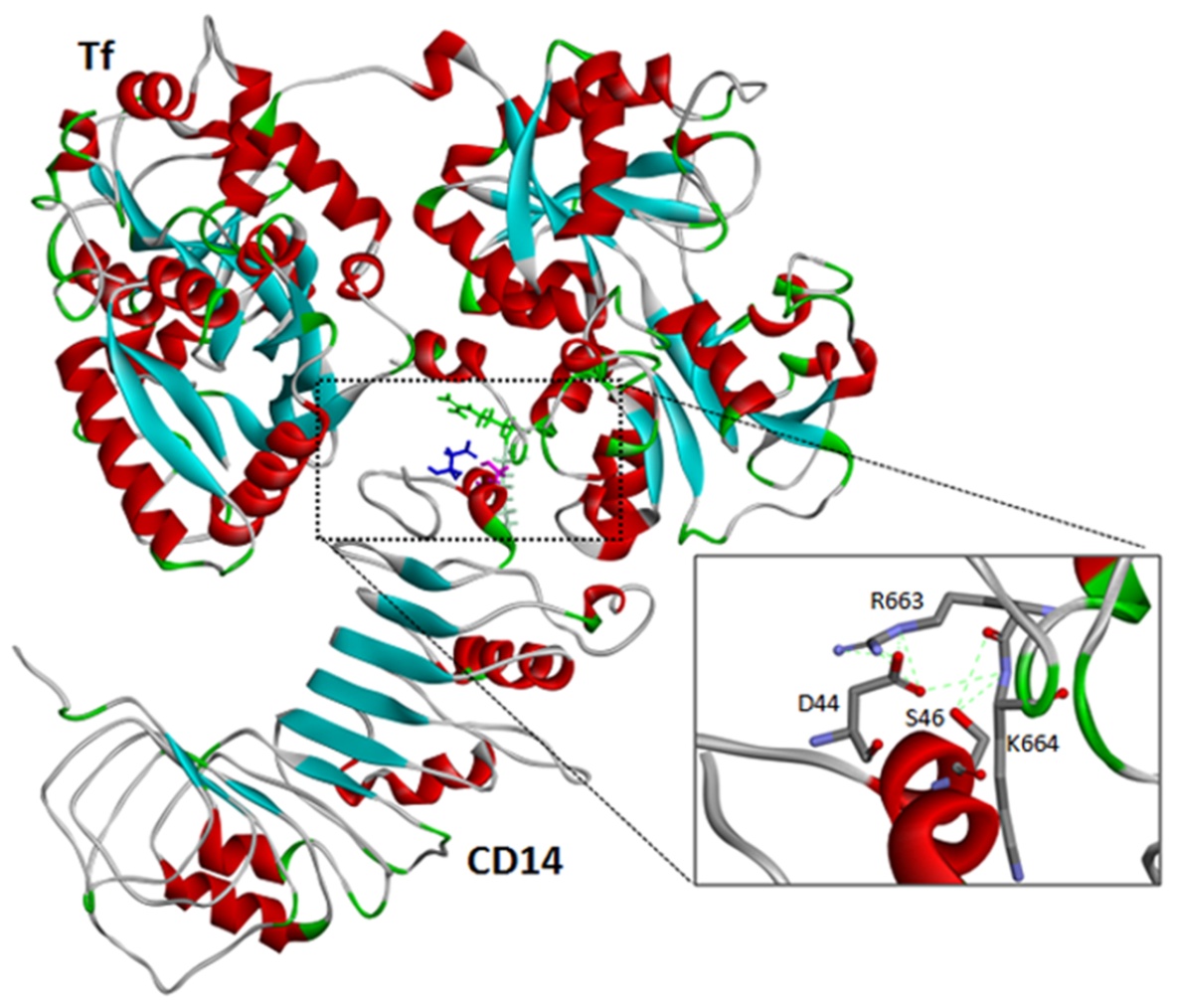
**

**Figure S7. Interaction of transferrin with CD14 by molecular docking.** 3D structure represents complex of transferrin and CD14. Different colors represent various secondary structure types, green: turn; red: helix; white: coil; blue: sheet. Dotted green lines in zoomed box indicate hydrogen bonds of labeled critical residues.

**
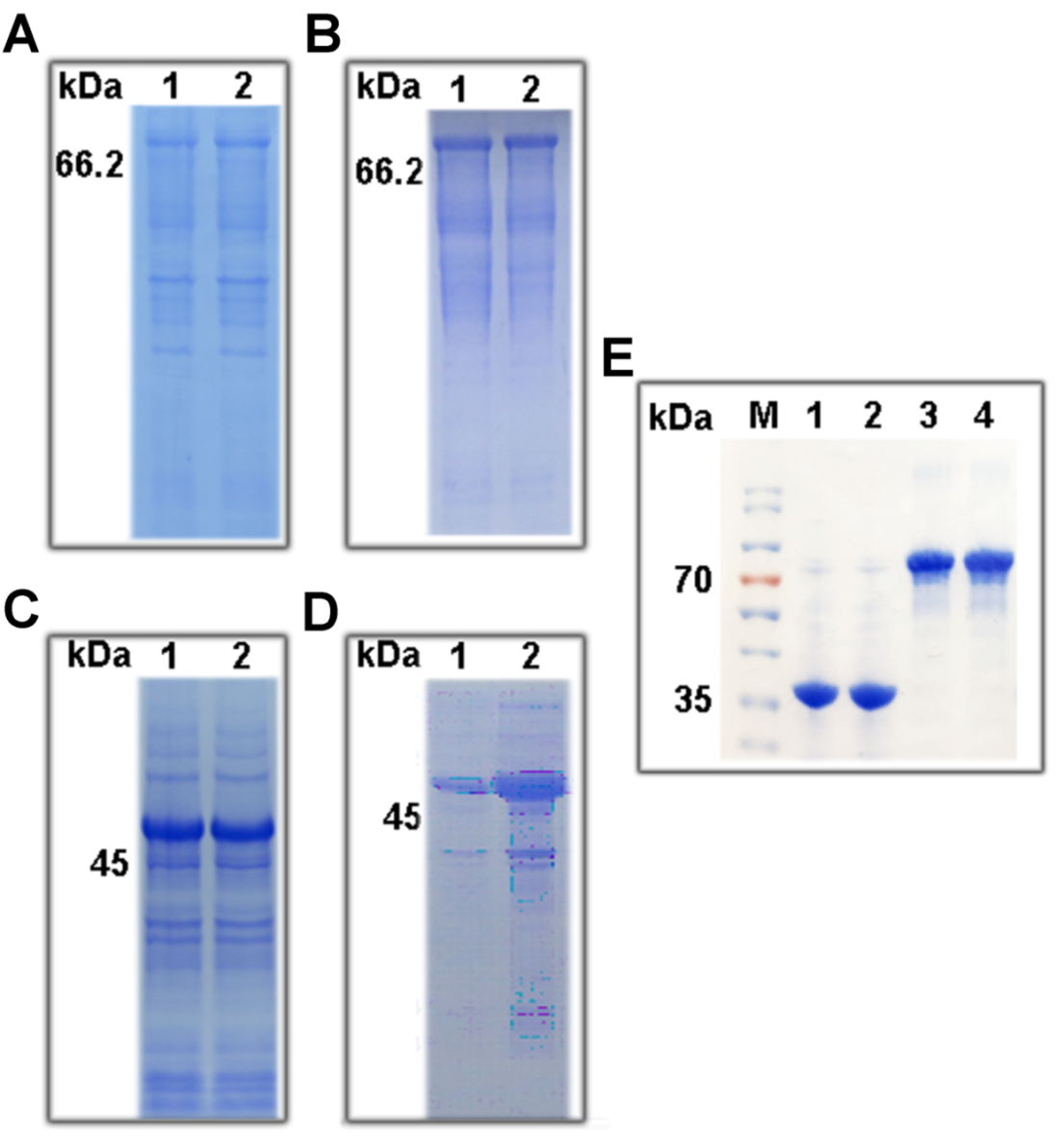
**

**Figure S8. Recombinant expression of transferrin, CD14, and their mutants. (A)** SDS-PAGE analysis of production of fused wild-type transferrin and its mutant after IPTG induction. Lane 1: wild-type transferrin, induced; Lane 2: transferrin mutant, induced. **(B)** Bound fusion protein of Ni^2+^ affinity chromatography column. Lane 1: fusion protein of wild-type transferrin; Lane 2: fusion protein of transferrin mutant. **(C)** SDS-PAGE analysis of production of fused wild-type CD14 and its mutant after IPTG induction. Lane 1: wild-type CD14, non-induced; Lane 2: CD14 mutant, induced. **(D)** Bound fusion protein of Ni^2+^ affinity chromatography column. Lane 1: fusion protein of wild-type CD14; Lane 2: fusion protein of CD14 mutant. **(E)** Purified transferrin, CD14, and their mutants. M: protein marker; Lane 1: purified wild-type CD14; Lane 2: purified CD14 mutant; Lane 3: purified wild-type transferrin; Lane 4: purified transferrin mutant.


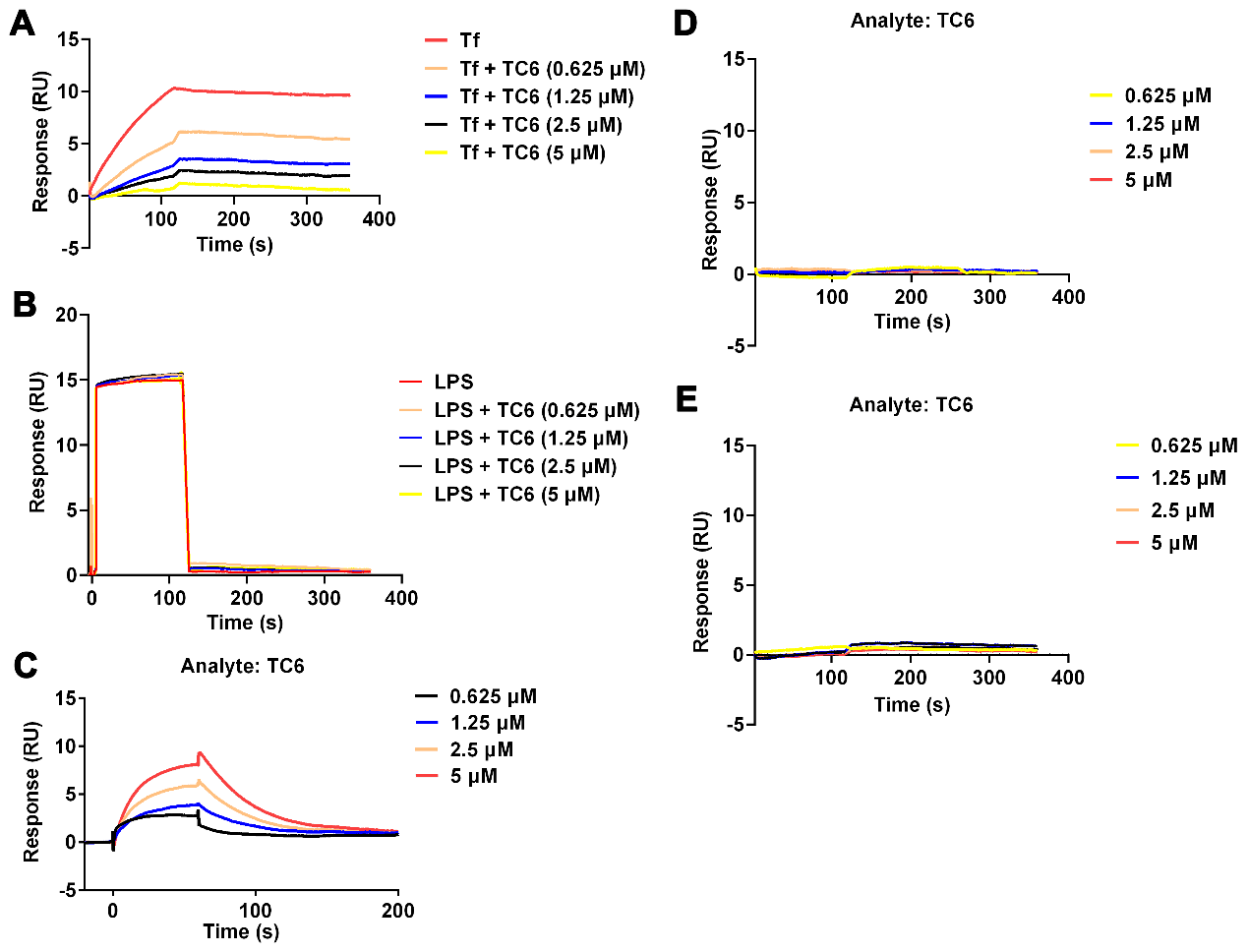


**Figure S9. TC6 inhibited transferrin-CD14 interaction without affecting LPS’s binding to CD14. (A)** TC6’s inhibition on the interaction between transferrin and CD14 by SPR analysis. **(B)** TC6 shows no effect on LPS-CD14 interaction. SPR analysis of the interaction between TC6 and transferrin **(C)**. TC6 shows no interaction with CD14 **(D)** or LPS **(E)**. Tf: transferrin.


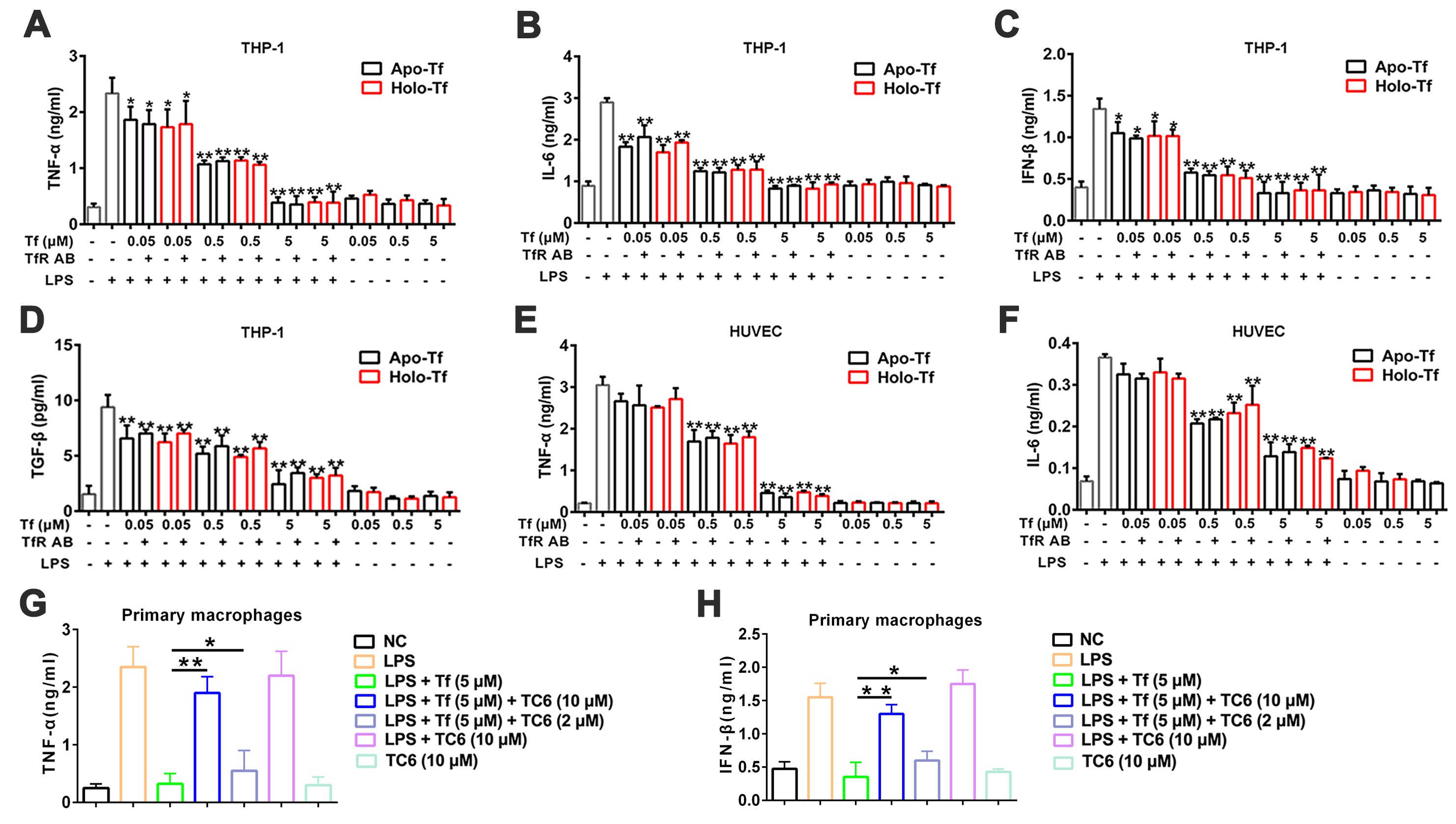


**Figure S10. Transferrin inhibits production of cytokines and type I interferon induced by LPS in immune and non-immune cells.** Human monocytic cells (THP-1) and human umbilical vein endothelial cells (HUVECs) were stimulated in presence or absence of apo- or holo-transferrin by LPS for 8 h. Some groups of cells were first incubated with anti-transferrin receptor antibody (TfR AB, 10 μg/ml) for 30 min. Effects of apo- or holo-transferrin on TNF-α, IL-6, IFN-β, or TGF-β production induced by LPS in THP-1 **(A-D)** or HUVECs **(E-F)** are shown. Effect of TC6 on the blockage of transferrin inhibited TNF-α **(G)** or IFN-β **(H)** production induced by LPS in primary macrophages are shown. Data represent means ± SD of five independent experiments, **p*< 0.05, ***p*< 0.01 by one-way ANOVA with Dunnett’s *post-hoc* test. Tf: transferrin.

**
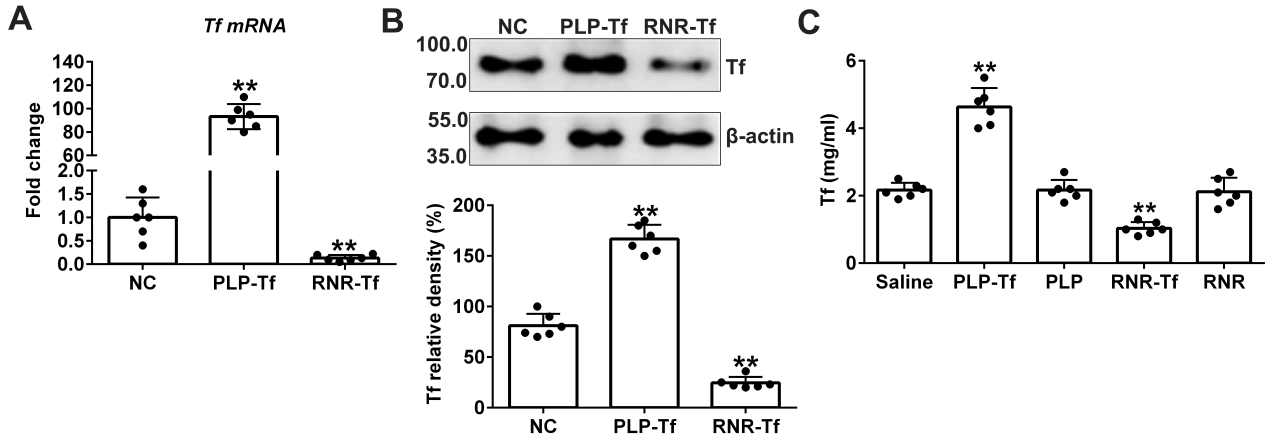
**

**Figure S11. Construction of transferrin overexpression or knockdown vectors. (A)** *Transferrin* mRNA levels of BNL CL.2 cells after transfection of overexpression or knockdown plasmid of transferrin determined by qRT-PCR. **(B)** Transferrin levels in BNL CL.2 cells determined by Western blotting (top, Lane 1: control (Saline), Lane 2: overexpression (PLP-Tf), Lane 3: knockdown (RNR-Tf)). Quantification of Western blots is also shown (bottom). Data represent means ± SD of six independent experiments, ***p* < 0.01 by unpaired *t*-test. Tf: transferrin. **(C)** Plasma concentrations of transferrin in three groups of C57BL/6J mice (PLP-Tf and its blank PLP, RNR-Tf and its blank RNR, and control mice (Saline)). Data represent means ± SD (n = 6), ***p*< 0.01 by unpaired *t*-test. Tf: transferrin.


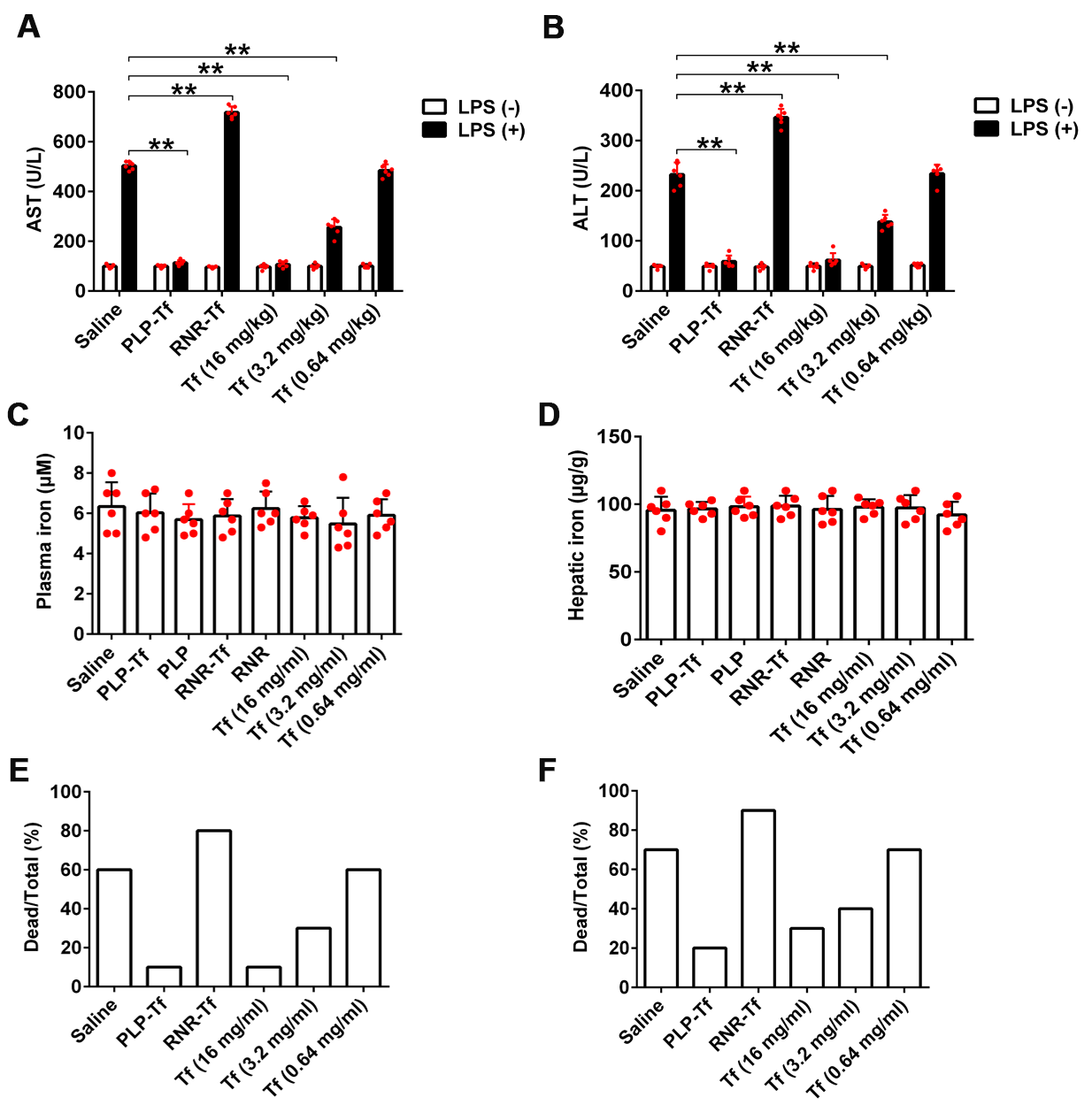


**Figure S12. Transferrin shows protective effects on injuries induced by LPS or bacterial infection without effecting iron level in mice. A & B:** Transferrin inhibited the elevation of alanine transaminase (ALT, **A**) and aspartate aminotransferase (AST, **B**) induced by LPS (750 μg/kg, intravenous injection after transferrin treatment for 20 min through the tail vein). **C & D**: Transferrin overexpression, knock-down or exogenous transferrin administration had no effects on iron levels of plasma **(C)** and liver tissue **(D)**. **E & F**: Transferrin decreased mouse lethality caused by intravenously injected LPS (15 mg/kg, **E**) or [*E.*](javascript:void(0);) [*coli*](javascript:void(0);) (10^9^ CFU/kg, **F**). Mouse groups included transferrin overexpression (PLP-Tf), knockdown (RNR-Tf), or their blank (PLP and RNR). Data represent means ± SD (n = 6), ***p*< 0.01 by one-way ANOVA with Dunnett’s *post-hoc* test. Tf: transferrin.


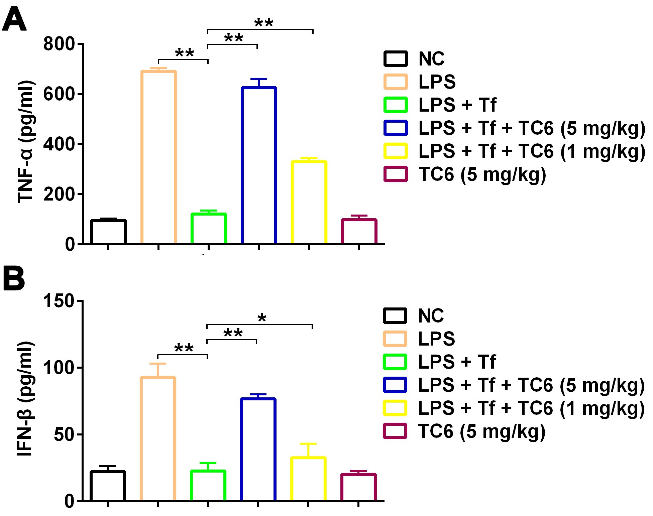


**Figure S13. TC6 inhibits the transferrin’s inhibition on plasma TNF-α and IFN-β secretion in vivo.** LPS (750 μg/kg) was injected into the tail vein of different concentrations of TC6 (1 or 5 mg/kg) treated mouse groups to induce an [inflammatory](javascript:void(0);) [response](javascript:void(0);) for 2 h. Plasma TNF-α **(A)** and IFN-β **(B)** level was determined by ELISA. Data represent means ± SD (n = 6), **p*< 0.05, ***p*< 0.01 by one-way ANOVA with Dunnett’s *post-hoc* test. Tf: transferrin.


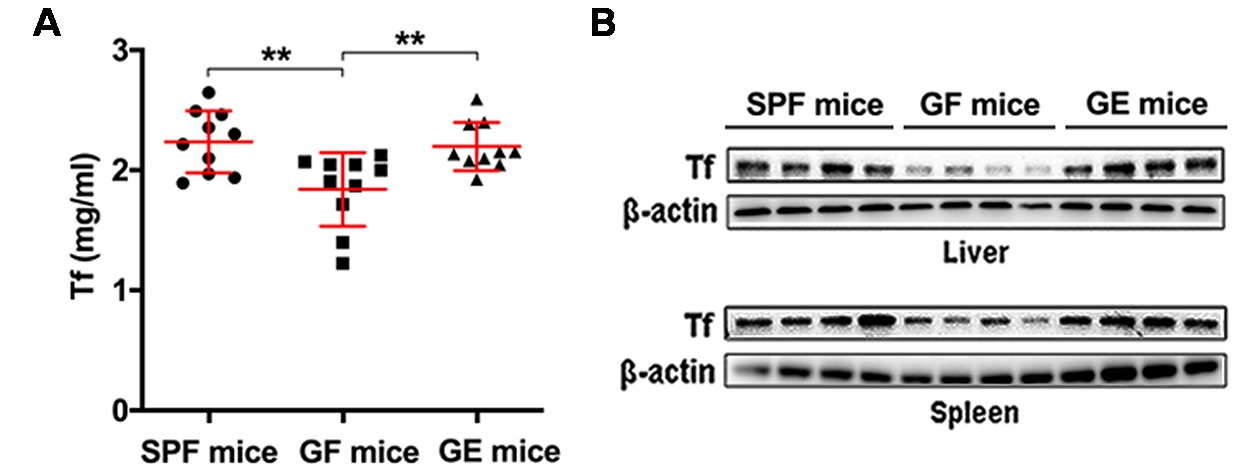


**Figure S14. Transferrin is decreased in germ-free mice. (A)** Transferrin concentration in plasma of germ-free (GF) and specific pathogen-free (SPF, control) mice determined by ELISA. Data represent means ± SD (n = 10), **p*< 0.05, ***p*< 0.01 by unpaired *t-*test. **(B)** Western blotting analyses of transferrin levels in protein extracts from liver and spleen of SPF and GF mice. [β](https://www.baidu.com/link?url=4TNZgg20byrlsiww7hxGBbFzbOn47fFIbJmiM3wNQ-asZvnk54ANh8-rybjWo1LUu26KsYGTBRQuOPg28rp35FXGk9gZ0a72RcrwzBky1N_&wd=&eqid=a1b0b1240004082b000000055b42bb94)-actin was used as the control.


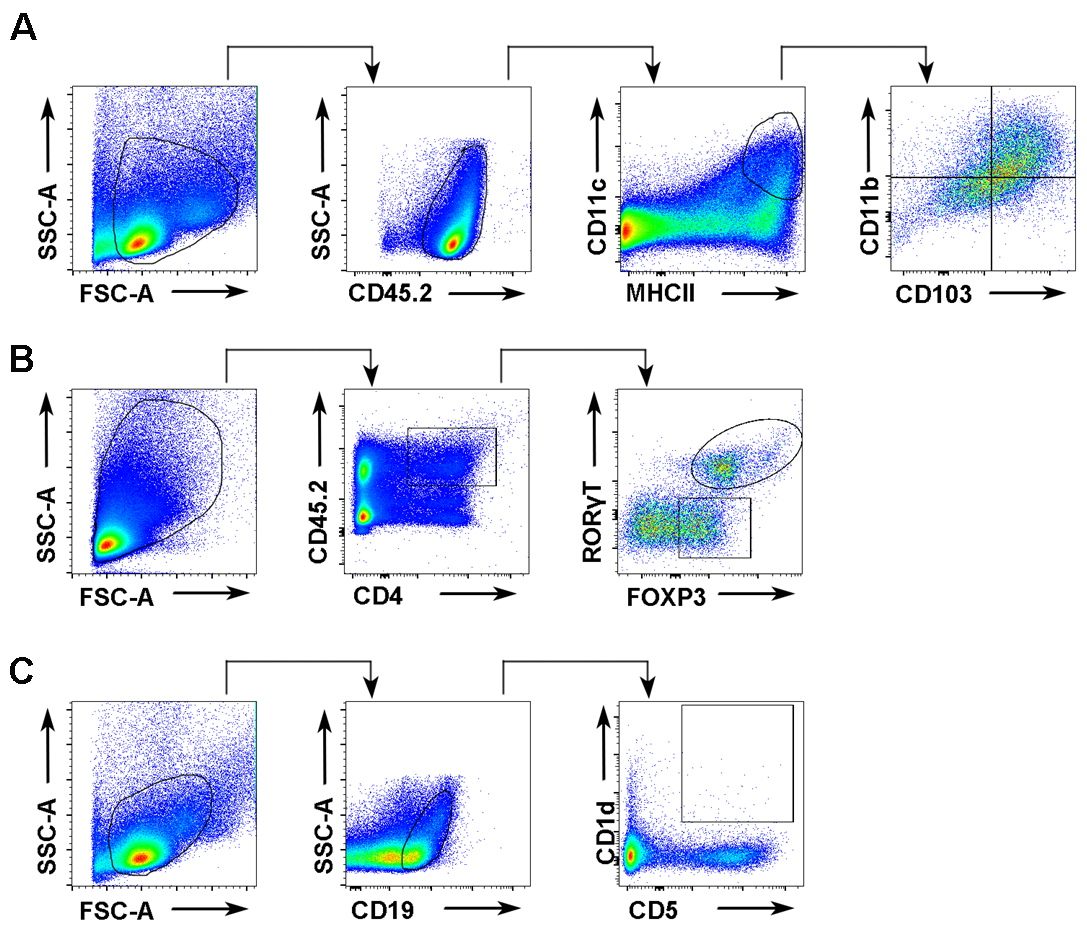


**Figure S15.** **Gating strategy to analyze intestinal tissues and lymph nodes of DCs, Tregs, and Bregs subsets. (A)** Cells isolated from gut tissues and gut-draining lymph nodes were stained with anti-45.2, anti-MHCII, anti-CD11c, anti-CD11b, and anti-CD103 antibodies. CD103^+^ DCs were identified as CD45.2^+^MHCII^+^CD11c^+^CD11b^-^CD103^+^, double positive (DP) DCs were identified as CD45.2^+^MHCII^+^CD11c^+^CD11b^+^CD103^+^, and CD11b^+^ DCs were identified as CD45.2^+^MHCII^+^CD11c^+^CD11b^+^CD103^-^. **(B)** Cells isolated from gut tissues and gut-draining lymph nodes were stained with anti-45.2, anti-CD4, anti-Foxp3, and anti-RORγT antibodies. Foxp3^+^Tregs were identified as CD45.2^+^CD4^+^Foxp3^+^RORγT^-^ and Foxp3^+^RORγT^+^Tregs were identified as CD45.2^+^CD4^+^Foxp3^+^RORγT^+^. **(C)** Bregs were identified as CD19^+^CD5^+^CD1d^+^ from gut tissues and gut-draining lymph nodes.


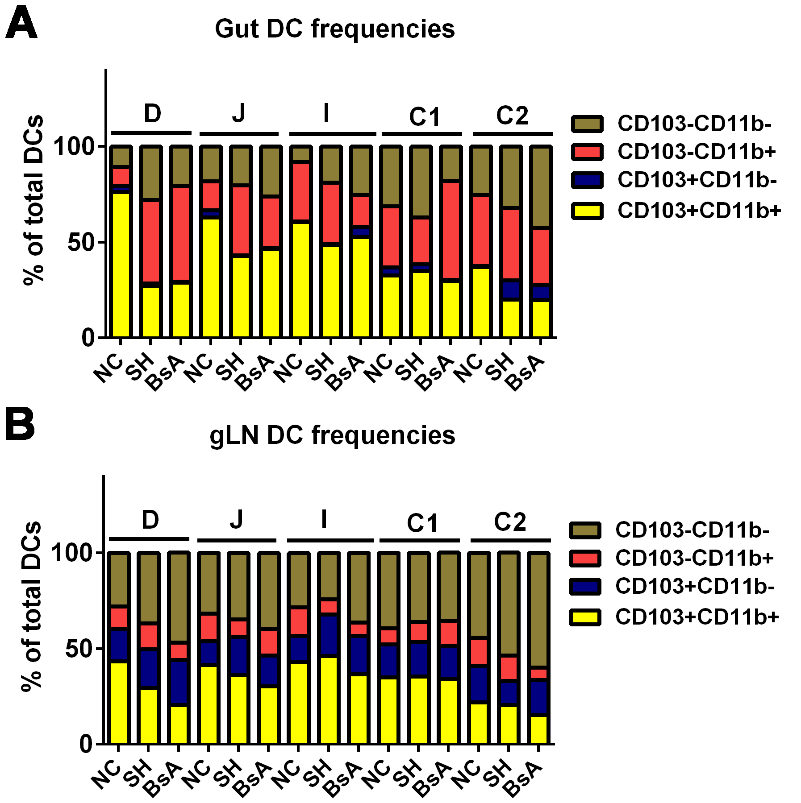


**Figure S16. Both transferrin-knockdown and broad-spectrum antibiotic treatment decreased the frequency, number, and proliferation of CD103^+^DC cells in mouse gut (A) and gut-draining lymph nodes (B).** Quantification analysis of CD103^+^DC cells in different segments (duodenum (D), jejunum (J), ileum (I), caecum (C1), and colon (C2)) of gut tissue and gut-draining lymph nodes (gLN) in ‘**Fig. 6E and F**’. NC: SPF mice, SH: transferrin knockdown SPF mice, BsA: Broad-spectrum antibiotic-treated mice**.**


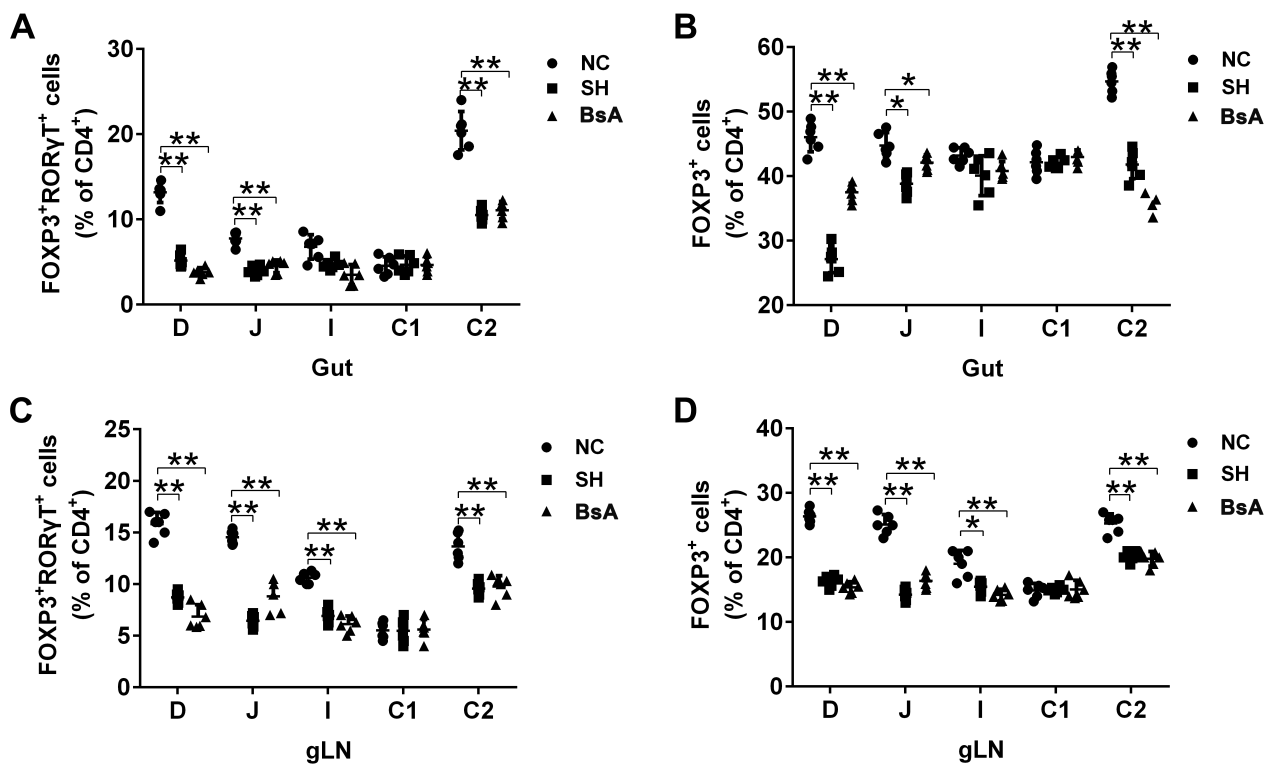


**Figure S17. Both transferrin-knockdown and broad-spectrum antibiotic treatment decreased the frequency, number, and proliferation of Foxp3^+^ and RORγT^+^ Tregs in mouse gut (A & B) and gut-draining lymph nodes (C & D).** Quantification analysis of Foxp3^+^ and Foxp3^+^ROROT^+^Tregs in gut tissue and gut-draining lymph nodes (gLN) in ‘**Fig. 6G and H**’. NC: SPF mice, SH: transferrin knockdown SPF mice, BsA: Broad-spectrum antibiotic-treated mice**.** Data represent means ± SD (n = 6), **p*< 0.05, ***p*< 0.01 by unpaired *t-*test.


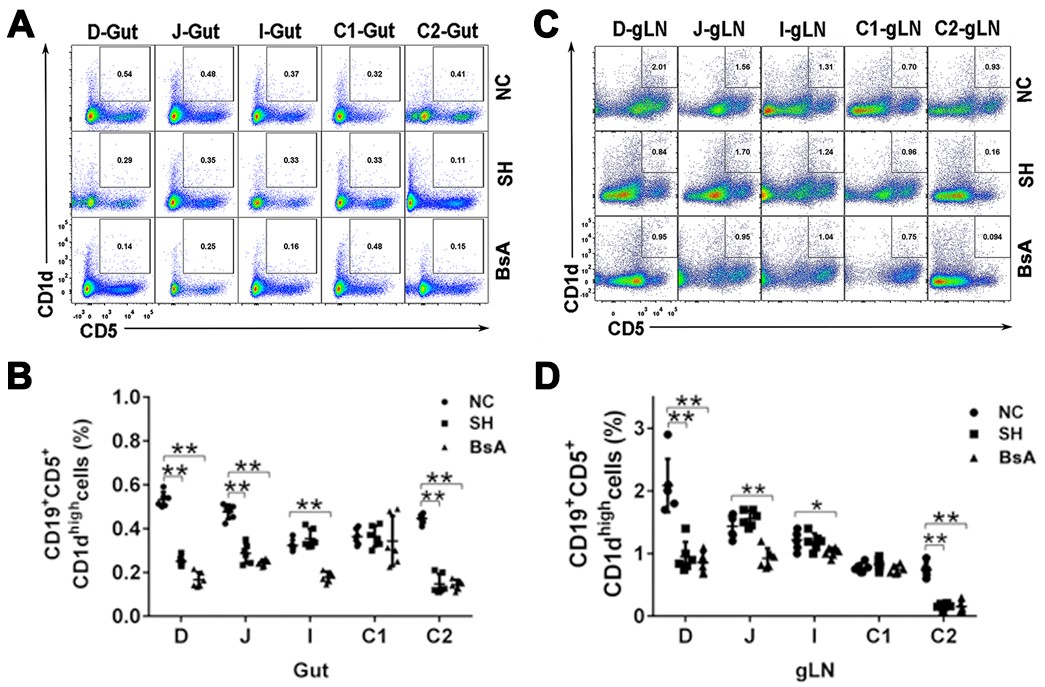


**Figure S18. Both transferrin-knockdown and broad-spectrum antibiotic treatment decreased the frequency, number, and proliferation of CD19^+^CD5^+^ Bregs in mouse gut (A & B) and gut-draining lymph nodes (C & D).** Bregs in gut tissue and gLN of all mice groups were characterized as CD19^+^ and further subdivided into CD5^+^CD1d^+^ (Fig. S15C). NC: SPF mice, SH: transferrin knockdown SPF mice, BsA: Broad-spectrum antibiotic-treated mice**.** Data represent means ± SD (n = 6), **p*< 0.05, ***p*< 0.01 by unpaired *t-*test.


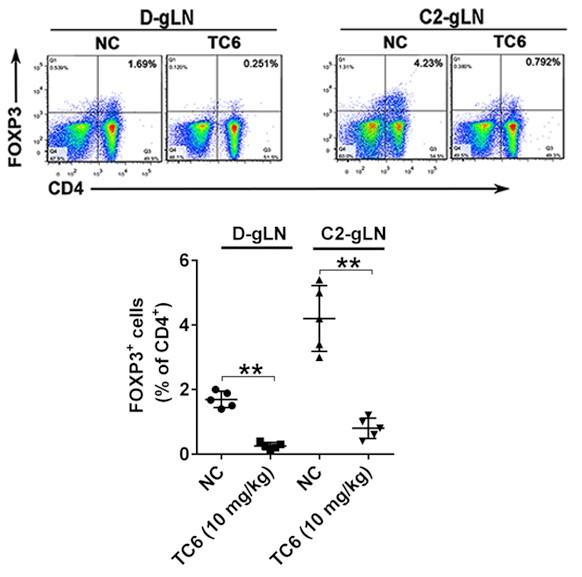


**Figure S19. TC6 treatment decreased the frequency, number, and proliferation of Foxp3^+^ Tregs in mouse gut-draining lymph nodes.** Foxp3^+^ Tregs in D- and C2-gLN of TC6 (10 mg/kg)-treated mice were analyzed (top) and quantification analysis of Foxp3^+^ Tregs in gut-draining lymph nodes (gLN) are shown (bottom). NC: SPF mice. Data represent means ± SD (n = 5), **p*< 0.05, ***p*< 0.01 by unpaired *t-*test.


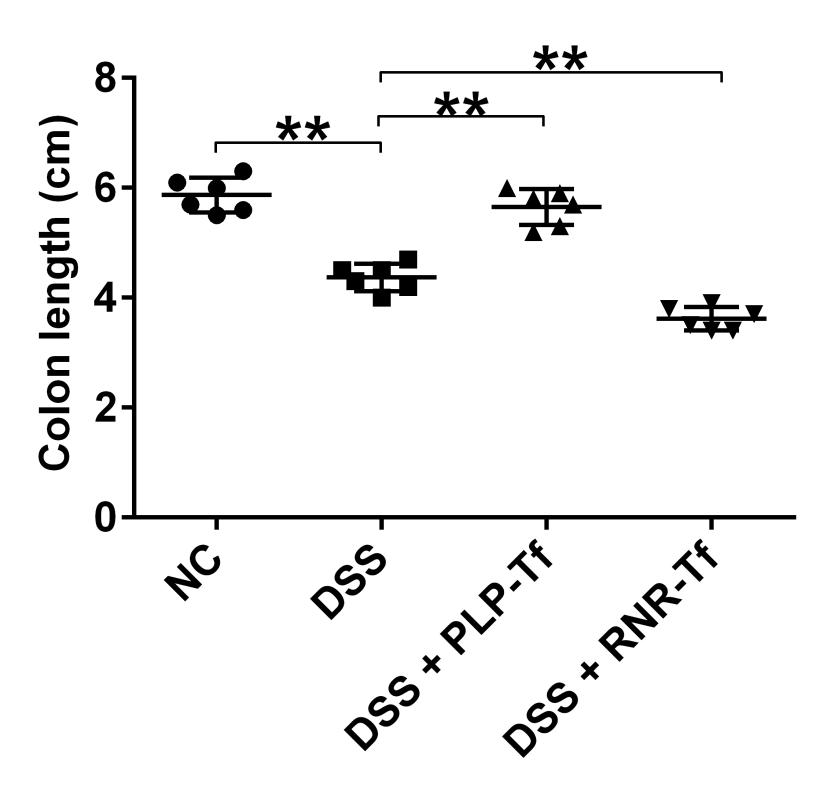


**Figure S20. Transferrin overexpression and knockdown inhibited and promoted the decrease of mouse colon length induced by DSS, respectively.** Quantification analysis of colon length in mouse colitis model induced by 5% DSS in ‘**Fig. 7E**’. NC: SPF mice, PLP-Tf: transferrin overexpression mice; RNR-Tf: transferrin knockdown mice. Data represent means ± SD (n = 6), **p*< 0.05, ***p*< 0.01 by unpaired *t*-test.


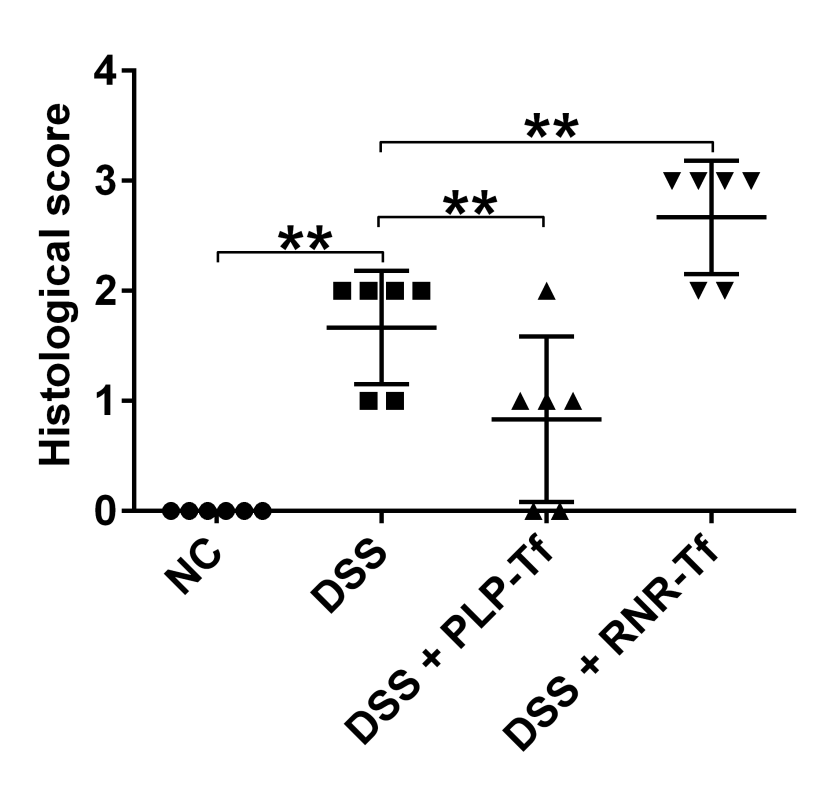


**Figure S21.** **Transferrin overexpression and knockdown inhibited and exacerbated mouse colon histological injury induced by DSS, respectively.** Quantification analysis of 5% DSS-induced mouse colon histopathological injury stained by hematoxylin and eosin in ‘**Fig. 7F**’. NC: SPF mice, PLP-Tf: transferrin overexpression mice; RNR-Tf: transferrin knockdown mice. Data represent means ± SD (n = 6), **p*< 0.05, ***p*< 0.01 by unpaired *t*-test.

**
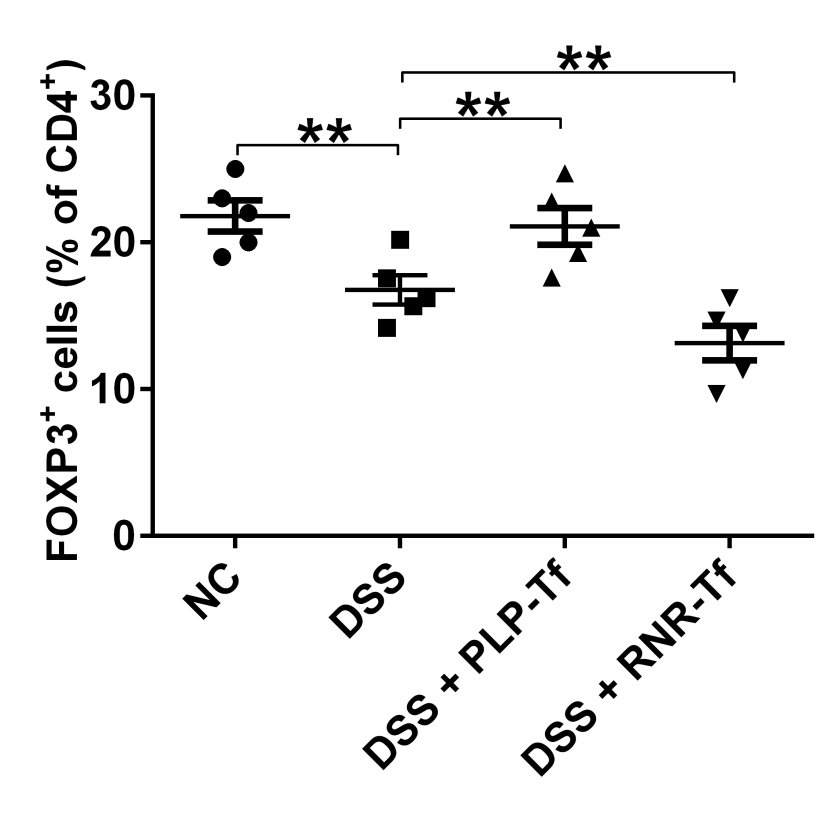
**

**Figure S22. Transferrin overexpression and knockdown inhibited and promoted the decrease of Foxp3^+^Treg cells in mouse gut-draining lymph nodes induced by DSS, respectively.** Quantification analysis of Foxp3^+^Treg cells in gut-draining lymph nodes of mouse DSS model in ‘**Fig. 7G**’. NC: SPF mice, PLP-Tf: transferrin overexpression mice; RNR-Tf: transferrin knockdown mice. Data represent means ± SD (n = 5), ***p*< 0.01 by unpaired *t*-test.


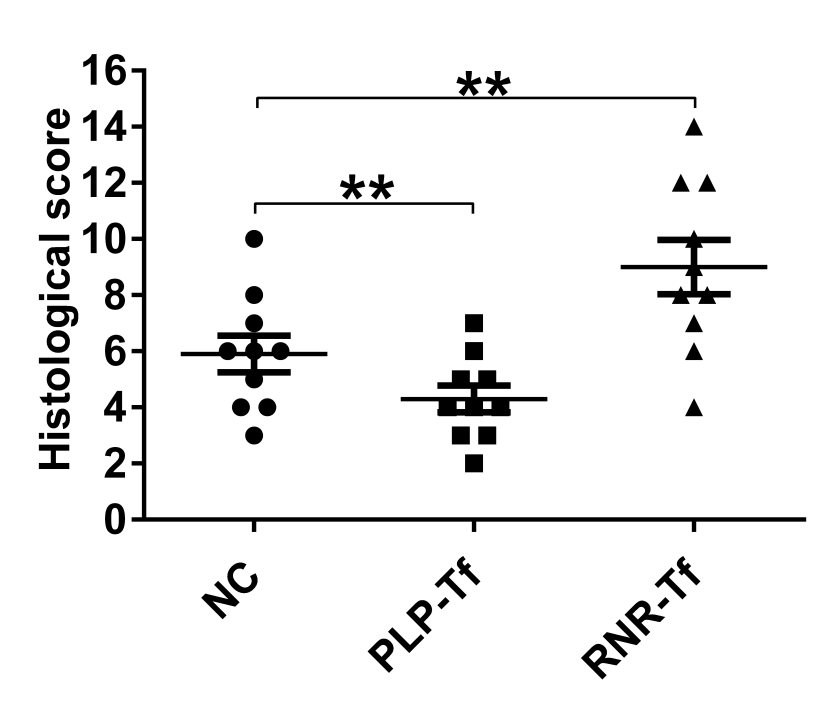


**Figure S23. Transferrin overexpression and knockdown inhibited and exacerbated colon histological injury in TCRα KO mice, respectively.** Quantification analysis of colon histopathological injury stained by hematoxylin and eosinin TCRα KO mice in ‘**Fig. 7H**’. NC: SPF mice, PLP-Tf: transferrin overexpression mice; RNR-Tf: transferrin knockdown mice. Data represent means ± SD (n = 6), **p*< 0.05, ***p*< 0.01 by unpaired *t*-test.


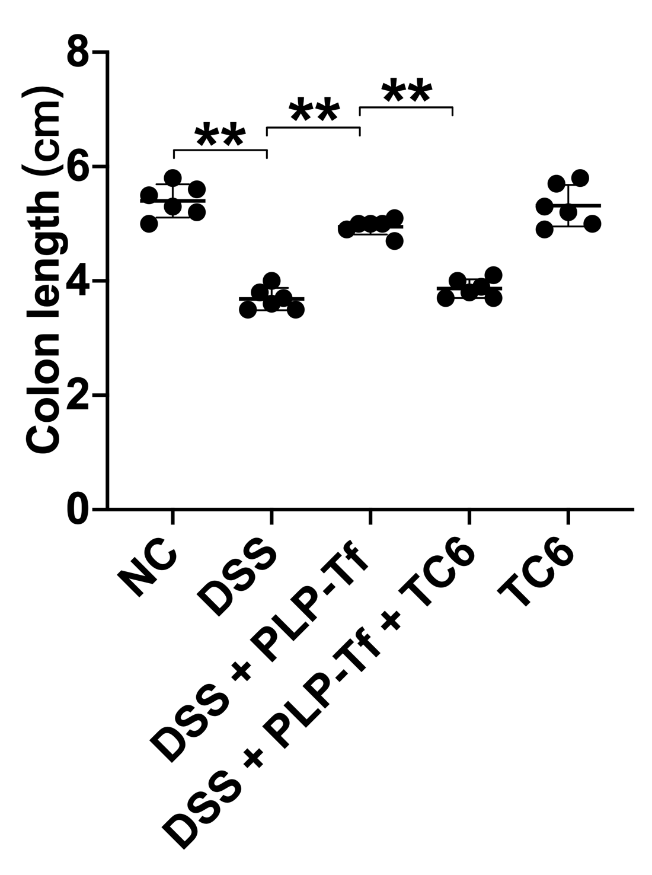


**Figure S24. TC6 treatment reversed the inhibition of colon length shortening by transferrin.** Quantification analysis of colon length in mouse colitis model induced by 5% DSS in ‘**Fig. 7J**’. Data represent means ± SD (n = 6), **p*< 0.05, ***p*< 0.01 by unpaired *t*-test.

**Supplementary References**

1. X. Tang *et al.*, Transferrin plays a central role in coagulation balance by interacting with clotting factors. *Cell Res* **30**, 119-132 (2020).

2. Y. Yang *et al.*, Amygdalin reduces lipopolysaccharide-induced chronic liver injury in rats by down-regulating PI3K/AKT, JAK2/STAT3 and NF-kappaB signalling pathways. *Artif Cells Nanomed Biotechnol* **47**, 2688-2697 (2019).

3. D. Esterházy *et al.*, Compartmentalized gut lymph node drainage dictates adaptive immune responses. *Nature* **569**, 126-+ (2019).

4. A. Mortha *et al.*, Microbiota-Dependent Crosstalk Between Macrophages and ILC3 Promotes Intestinal Homeostasis. *Science* **343**, 1477-+ (2014).

5. L. X. Sang *et al.*, Sodium selenite ameliorates dextran sulfate sodium-induced chronic colitis in mice by decreasing Th1, Th17, and gamma delta T and increasing CD4(+) CD25(+) regulatory T-cell responses. *World J Gastroentero* **23**, 3850-3863 (2017).

6. K. Sugimoto *et al.*, Inducible IL-12-Producing B cells regulate Th2-mediated intestinal inflammation. *Gastroenterology* **133**, 124-136 (2007).

7. R. S. Blackwood, R. P. Tarara, K. L. Christe, A. Spinner, N. W. Lerche, Effects of the macrolide drug tylosin on chronic diarrhea in rhesus macaques (Macaca mulatta). *Comp Med* **58**, 81-87 (2008).
